# Graphene-confined ultrafast radiant heating for high-loading subnanometer metal cluster catalysts

**DOI:** 10.1093/nsr/nwad081

**Published:** 2023-03-23

**Authors:** Ye-Chuang Han, Jun Yi, Beibei Pang, Ning Wang, Xu-Cheng Li, Tao Yao, Kostya S Novoselov, Zhong-Qun Tian

**Affiliations:** State Key Laboratory of Physical Chemistry of Solid Surfaces, College of Chemistry and Chemical Engineering, Graphene Industry and Engineering Research Institute, School of Electronic Science and Engineering, Xiamen University, Xiamen 361005, China; Innovation Laboratory for Sciences and Technologies of Energy Materials of Fujian Province (IKKEM), Xiamen 361005, China; State Key Laboratory of Physical Chemistry of Solid Surfaces, College of Chemistry and Chemical Engineering, Graphene Industry and Engineering Research Institute, School of Electronic Science and Engineering, Xiamen University, Xiamen 361005, China; Innovation Laboratory for Sciences and Technologies of Energy Materials of Fujian Province (IKKEM), Xiamen 361005, China; National Synchrotron Radiation Laboratory, University of Science and Technology of China, Hefei 230029, China; Faculty of Environment and Life, Beijing University of Technology, Beijing 100124, China; State Key Laboratory of Physical Chemistry of Solid Surfaces, College of Chemistry and Chemical Engineering, Graphene Industry and Engineering Research Institute, School of Electronic Science and Engineering, Xiamen University, Xiamen 361005, China; Innovation Laboratory for Sciences and Technologies of Energy Materials of Fujian Province (IKKEM), Xiamen 361005, China; National Synchrotron Radiation Laboratory, University of Science and Technology of China, Hefei 230029, China; State Key Laboratory of Physical Chemistry of Solid Surfaces, College of Chemistry and Chemical Engineering, Graphene Industry and Engineering Research Institute, School of Electronic Science and Engineering, Xiamen University, Xiamen 361005, China; Institute for Functional Intelligent Materials, National University of Singapore, Singapore 117544, Singapore; State Key Laboratory of Physical Chemistry of Solid Surfaces, College of Chemistry and Chemical Engineering, Graphene Industry and Engineering Research Institute, School of Electronic Science and Engineering, Xiamen University, Xiamen 361005, China; Innovation Laboratory for Sciences and Technologies of Energy Materials of Fujian Province (IKKEM), Xiamen 361005, China

**Keywords:** photothermal conversion, ultrafast radiant heating, graphene-confined, high-loading metal clusters, solid catalysts

## Abstract

Thermally activated ultrafast diffusion, collision and combination of metal atoms comprise the fundamental processes of synthesizing burgeoning subnanometer metal clusters for diverse applications. However, so far, no method has allowed the kinetically controllable synthesis of subnanometer metal clusters without compromising metal loading. Herein, we have developed, for the first time, a graphene-confined ultrafast radiant heating (GCURH) method for the synthesis of high-loading metal cluster catalysts in microseconds, where the impermeable and flexible graphene acts as a diffusion-constrained nanoreactor for high-temperature reactions. Originating from graphene-mediated ultrafast and efficient laser-to-thermal conversion, the GCURH method is capable of providing a record-high heating and cooling rate of ∼10^9^°C/s and a peak temperature above 2000°C, and the diffusion of thermally activated atoms is spatially limited within the confinement of the graphene nanoreactor. As a result, due to the kinetics-dominant and diffusion-constrained condition provided by GCURH, subnanometer Co cluster catalysts with high metal loading up to 27.1 wt% have been synthesized by pyrolyzing a Co-based metal-organic framework (MOF) in microseconds, representing one of the highest size-loading combinations and the quickest rate for MOF pyrolysis in the reported literature. The obtained Co cluster catalyst not only exhibits an extraordinary activity similar to that of most modern multicomponent noble metal counterparts in the electrocatalytic oxygen evolution reaction, but is also highly convenient for catalyst recycling and refining due to its single metal component. Such a novel GCURH technique paves the way for the kinetically regulated, limited diffusion distance of thermally activated atoms, which in turn provides enormous opportunities for the development of sophisticated and environmentally sustainable metal cluster catalysts.

## Teaser

In order to overcome the trade-off between ultrasmall size and high loading in synthesizing high-performance metal cluster catalysts, a novel graphene-confined ultrafast radiant heating (GCURH) method is developed.

## INTRODUCTION

The controlled formation of a variety of predesigned metallic nanostructures through the selection of optimal kinetic circumstances has been the primary focus of materials scientists for a very long time [1,2]. In particular, subnanometer metal clusters, which are formed by a collection of metal atoms, are long and highly sought-after structures, mainly for their catalytic properties [3–5]. The size-dependent geometric structure and unique electronic properties of such clusters offer them a diverse array of surface sites for the effective adsorption, activation and transformation of reactant molecules in multistep tandem reactions [6−12]. Consequently, the field of metal cluster catalysts is flourishing and shows tremendous promise in both traditionally remarkable thermocatalysis (e.g. ammonia synthesis) [6,7] and recently flourishing electrocatalysis (e.g. water splitting) [8]. The most prevalent method for producing metal cluster catalysts is high-temperature reduction, which typically involves hours of program-controlled heating of metal-ion-impregnated solid support (e.g. alumina, silica, nanocarbons) [9]. To prevent the coalescence of adjacent metal clusters caused by arduous high-temperature treatment, the metal loading is typically kept at a relatively low level (≤1 wt%) [10]. However, catalysts with such a low metal loading inevitably suffer from a limited number of active sites, which ultimately limits their effectiveness in heterogeneous catalysis [11,12]. Therefore, the controlled preparation of high-loading metal cluster catalysts remains a formidable challenge, which is crucial for both future laboratory and industrial applications.

To resolve these challenges and thereby meet the increasing demand for efficient synthesis, extensive effort has been devoted to the development of rapid heating technologies, such as microwave heating, Joule heating and laser heating [13–16]. In general, microwave heating and Joule heating can produce high-temperature pulses with a heating rate of up to ∼10^5^°C/s on the second (s) or millisecond (ms) scale. The fabrication of metal nanocatalysts has advanced remarkably as a result of these innovative methods [17−19]. In a typical Joule-heating or microwave-heating procedure, three key elements contribute to smaller particle size distributions of metal nanocatalysts: short pulse duration (typically on the ms scale), low metal loading and defect-rich support. If the optimal circumstances are not fulfilled, the reduced metal atoms would be coalescing or sintered into larger nanoparticles. The fast growth kinetics of thermally activated and dynamically diffused metal atoms is the most frequent cause of such occurrences [20,21]. Therefore, the ms scale of a high-temperature pulse is not fast enough to prevent the coalescence of densely dispersed metal atoms, and nanoparticles with an average size above 1 nm are typically produced [19,22]. Laser heating, such as direct nanosecond (ns) laser irradiation, is capable of producing sub-millisecond high-temperature pulses with a heating rate above 10^5^°C/s [23]. However, the intense photothermal effect of an ns laser would lead to the evaporation and subsequent vigorous diffusion of metal species (atoms and clusters) and organic molecules in unrestricted space [24,25]. Consequently, the freely diffused metal species have more time and space to nucleate and grow into nanoparticles (typically larger than 3 nm) during the subsequent cooling process [26,27]. Accordingly, direct ns pulsed laser heating is inapplicable to the manufacture of high-loading metal cluster catalysts, as it cannot spatially limit the diffusion of thermally induced ultrafast diffusion, collision and combining of metal atoms.

**Figure 1.**
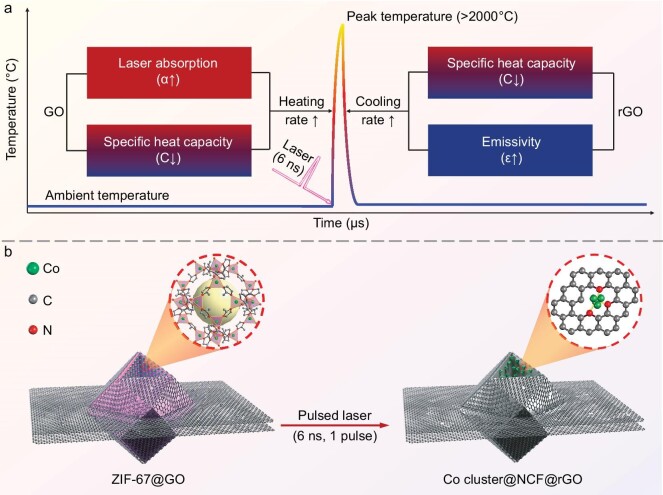
Nanosecond (ns)-laser-triggered and graphene-mediated GCURH of MOFs. (a) GO-mediated conversion of an incident ns laser to μs-scale IR thermal radiation. The high absorption and low specific heat capacity endow the optically thin GO sheets with a theoretically ultrafast heating rate when irradiated with sufficient laser energy. Similarly, the high thermal emissivity and low specific heat capacity of LI-GO endow it with an extraordinarily fast radiative cooling rate. (b) Constrained diffusion of thermally activated atoms/molecules caused by the confinement within the GO interlayer during the radiant pyrolysis of ZIF-67. The three intrinsic physicochemical properties, including high thermal stability, low permeability and high flexibility, enable GO to act as an interlayer confined nanoreactor for high-temperature reactions. The wavelength and pulse duration of the incident laser is 355 nm and 6 ns, respectively.

Herein, we develop a microsecond (μs)-scale graphene-confined ultrafast radiant heating (GCURH) method, which possesses a record-high heating (and cooling) rate of ∼10^9^°C/s and a peak temperature higher than 2000°C. During the typical heating process, graphene oxide (GO) can not only, ultrafast and efficiently, convert an incident laser pulse into μs-scale infrared (IR) thermal radiation for sample heating (Fig. 1a), but also spatially constrain the subsequent vigorous diffusion of thermally activated atoms or molecules within nearly impermeable GO or rGO (reduced GO) interlayers (Fig. 1b). Due to the diffusion-constrained μs-scale high-temperature conditions, and the well-designed GO-wrapped Co-based metal-organic framework (MOF) (ZIF-67) crystals that were prepared by a simple physical mixing in liquid and then a drop-cast procedure, we synthesized a high-loading Co cluster catalyst (27.1 wt%) via only one pulse of GCURH treatment within 40 μs, representing one of the highest size-loading combinations in the literature and the fastest rate for MOF pyrolysis. As demonstrated by the oxygen evolution reaction (OER) studies, the extraordinary activity of the obtained single-metal-component Co cluster catalyst is similar to that of advanced multicomponent noble metal counterparts, requiring an overpotential of only 231 mV to drive a current density of 10 mA cm^−2^. Moreover, the Co cluster catalyst remains stable at 100 mA cm^−2^ for 20 hours, and it has also shown itself to be remarkably convenient with regard to catalyst recycling and refining due to its single metal component when compared to its multicomponent counterparts. These results suggest that the novel GCURH technique provides a great opportunity to kinetically constrain the diffusion distance of thermally activated atoms, and for the development of advanced and sustainable metal cluster catalysts.

## RESULTS AND DISCUSSION

### GCURH in microseconds

**Figure 2.**
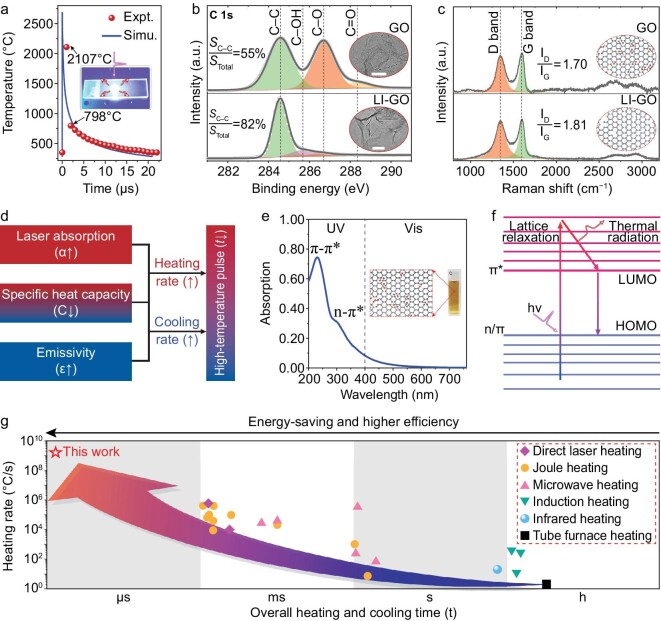
GCURH. (a) Temperature variation during one pulse of GCURH. Inset shows a digital photograph of GCURH. The incident laser energy is 150 mJ (wavelength, 355 nm; pulse duration, 6 ns). (b) C 1s surface XPS spectra and TEM images (inset, scale bar is 100 nm) of raw GO and LI-GO, showing the reduction of GO after laser irradiation. (c) Raman spectra and the corresponding structural scheme (inset) of raw GO and LI-GO, showing the well-maintained 2D structure and thermal stability of LI-GO after laser irradiation. (d) Rational construction of the laser-triggered high-temperature pulse, based on the unique physiochemical properties of GO; the three intrinsic properties, including efficient laser absorption, high emissivity and low specific heat capacity, endow GO with ultrafast heating and cooling rates during laser irradiation. (e) UV-Vis absorption of GO solution; the inset shows a digital photo of GO with a concentration of 0.2 mg mL^−1^ in an aqueous solution. (f) Schematic illustration for the GO-mediated ns laser heating and subsequent energy dissipation by radiation and thermal conduction. (g) Comparison of the heating rate and overall time (including heating and cooling) between the current study and reported literature.

For the controlled synthesis of high-loading metal cluster catalysts, we developed a μs-scale GCURH method, which possesses an instant heating/cooling rate of ∼10^9^°C/s and a peak temperature of >2000°C (experimental results in Fig. 2a, Fig. S1). The typical GCURH process consists of the following steps (Fig. S2): (i) the sample wrapped with GO was held tightly between two layers of quartz glass for laser energy absorption and conversion; (ii) the single laser pulse with a wavelength of 355 nm and a duration of 6 ns was used for irradiation and ultrafast heating of the GO film; (iii) the IR pyrometer equipped with a high-speed data-acquisition workstation was used for non-contact temperature measurement, and the sampling rate was one temperature point per μs. As a result, GO was heated to 2107°C in 1 μs at a heating rate of ∼2.1 × 10^9^°C/s by 150 mJ of single-pulse laser irradiation (the instantaneous laser power was 2.5 × 10^7^ watts).

In the subsequent cooling process, the temperature of laser-irradiated GO film (LI-GO, rGO in essence) dropped sharply from 2107°C to 798°C in 1 μs, with a cooling rate of 1.3 × 10^9^°C/s, remarkably faster than the cooling process over the next 20 μs, in which the temperature cooled from 798°C to 350°C with an average cooling rate of 2.2 × 10^7^°C/s. The experimentally observed significant difference in cooling rate, combined with our theoretical simulation (simulated results in Fig. 2a, Fig. S3), suggests the cooling process can be largely divided into the radiation-dominant stage (2107°C to 798°C; cooling rate, 1.3 × 10^9^°C/s) and the conduction-dominant stage (798°C to 350°C; cooling rate, 2.2 × 10^7^°C/s). Additional simulation details can be found in the Supplementary Methods. Nojeh *et al.* [28,29] reported a similar phenomenon, in which radiation heat transfer dominated the cooling of nanocarbon materials (e.g. graphene, carbon nanotube) at temperatures exceeding 1000 K (727°C). In addition, LI-GO is likely to convert into rGO via photo and thermal reduction while retaining its basic 2D morphology, as shown by X-ray photoelectron spectroscopy (XPS) and Raman spectra (Fig. 2b and c) [30,31], which is extremely useful in the construction of an impermeable and diffusion-constrained nanoreactor for high-temperature reactions (Fig. S4) [17,32].

To elucidate the process of GO-mediated transformation of an incident ultraviolet (UV) ns laser into μs-scale IR thermal radiation, the intrinsic physicochemical features of GO and its strong interactions with a UV laser were investigated further (Fig. 2d). First, atomically thin GO is highly absorptive over the UV spectral range due to the π to π* or n to π* transitions (Fig. 2e, Fig. S5). The high absorption and low specific heat capacity of GO, therefore, endow it with a theoretically ultrafast heating rate when injecting sufficient laser power [28]. Subsequently, the optically stimulated electrons then release energy by electron-electron scattering and electron-lattice interaction, the latter of which heats the GO lattice. Under ns laser irradiation, a thermal equilibrium is established between electron and lattice, and high-temperature GO dissipates thermal energy to the environment via black-body radiation and thermal conduction (Fig. 2f). At high temperatures, radiation dominates the rapid cooling of LI-GO. This process can be comprehended qualitatively using the Stefan-Boltzmann law [33,34]:


\begin{eqnarray*} C\frac{{dT}}{{dt}} = - \alpha \varepsilon \sigma \left( {{T}^4 - {T}_{amb.}^4} \right), \end{eqnarray*}

where *C* is the heat capacity per unit area, α is equal to 2 for radiation that occurs on both sides of graphene, *ϵ* is the emissivity, *σ* is the Stefan-Boltzmann constant and *T_amb__._* is the ambient temperature. The cooling rate or radiation energy density increases with the fourth power of temperature (∝*T*^4^). At elevated temperatures, radiation would, therefore, become the primary mode of energy release. In addition, the ratio of a material's emissivity (*ϵ*) to its specific heat capacity (*C*) determines the rate of its cooling. In other words, the high thermal emissivity (>95%) and low specific heat (near 0.7 J g^−1^ K^−1^) of graphene endow it with an extraordinarily fast radiative cooling rate, which is remarkably faster than that of other high-temperature-resistant materials (e.g. Cr, Mo and W) [28,35,36]. For example, the cooling rate of tungsten wire, a well-known luminous material with remarkable thermal radiation at elevated temperatures, is ∼6.0 × 10^3^°C/s when cooled from 2400°C to 800°C (Fig. S6), which is six orders of magnitude lower than that of its GO counterpart. In addition, the GCURH technique has the highest heating rate and shortest duration (including the heating and cooling processes) when compared to other known pulsed heating techniques, such as pulsed Joule heating, direct ns laser heating and microwave heating (Fig. 2g, Table S1). Overall, GO is a novel and highly promising platform for the construction of a temporally ultrafast and spatially constrained high-temperature pulsed physical field, and paves the way for the controlled synthesis of subnanometer metal cluster catalysts with high metal loading.

### Synthesis of subnanometer Co clusters

**Figure 3.**
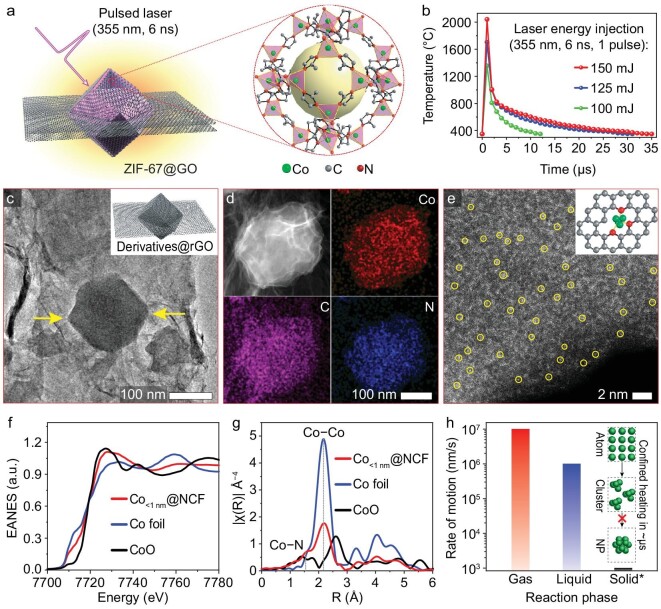
Synthesis of Co clusters by GCURH. (a) Schematic illustration for pyrolyzing ZIF-67 by GCURH. (b) Temperature variation during GCURH of ZIF-67 by adjusting incident laser energy. (c) TEM image and structural scheme (inset) of rGO wrapped ZIF-67 derivatives (Derivatives@rGO) prepared by irradiating 125 mJ of laser energy. (d) HAADF-STEM and corresponding EDX elemental mapping of Co_<1 nm_@NCF. (e) Spherical aberration-corrected HAADF-STEM image of Co_<1 nm_@NCF; the inset demonstrates the structural scheme for a Co cluster anchored on a carbon support by Co-N-C interfacial bonding. (f and g) XANES and EXAFS spectra of Co_<1 nm_@NCF, Co foil and CoO powder. (h) The Brownian motion of molecules or atoms in different phases, *near the melting temperature of the metal.

By GCURH treatment of a Co-based zeolitic imidazolate framework-67 precursor (ZIF-67, C_8_H_12_N_4_Co), high-loading subnanometer Co clusters wrapped in a nitrogen-doped carbon framework (Co_<1 nm_@NCF) were produced in the current study. We selected ZIF-67 as the precursor due to its crystallographically well-defined structure, atomically dispersed metal nodes and low production cost (Fig. 3a). In addition, high-temperature pyrolysis is becoming an increasingly attractive route for synthesizing MOF-derived functional materials (Fig. S7), and the Cambridge Structural Database lists >100 000 varieties of MOFs as possible precursors. Moreover, the transient heating and cooling rate during GCURH treatment of ZIF-67 was ∼10^9^°C/s, and the peak temperature window is adjustable between 1300°C and 2100°C by varying the injected laser energy (Fig. 3b).

After GCURH treatment with 125 mJ of single-pulse laser energy, ZIF-67 was heated to temperatures exceeding 1600°C (Fig. S8), wherein the pyrolyzed ZIF-67 was observed to retain its 3D morphology (Fig. 3c). Transmission electron microscopy (TEM) images and X-ray diffraction spectra show that no detectable metal nanoparticles were observed (Fig. S9). However, energy dispersive X-ray spectroscopy (EDX) elemental mapping shows that Co species dispersed in the derived nitrogen-doped carbon framework (NCF) with a high Co loading of 27.1 wt% or 7.3 at% (Fig. 3d, Fig. S10). These values are remarkably higher than those reported in the literature (Table S2). To discriminate Co species from the carbon matrix, spherical aberration-corrected high-angle annular dark-field scanning TEM (HAADF-STEM) was used. The HAADF-STEM image reveals many brilliant dots that correspond to Co atoms densely dispersed in the NCF with a diameter of ∼0.5 nm (Fig. 3e) [37], indicating that Co exists in the form of clusters and may be stabilized by interfacial bonding (inset of Fig. 3e). In addition, X-ray absorption near edge structure (XANES) and extended X-ray absorption fine structure (EXAFS) were employed to analyze the structures of Co in a Co_<1 nm_@NCF catalyst. Moreover, the Co K-edge XANES spectra show that the absorption edge of Co is positioned between CoO and Co foil, implying that the oxidation state of Co is between 0 and +2 (Fig. 3f). Furthermore, the EXAFS of Co_<1 nm_@NCF reveals two peaks at 2.19 Å and 1.54 Å, which are respectively attributed to Co−Co and Co−N (Fig. 3g, Figs S11 and S12, Table S3) [38], suggesting that the generated Co clusters were immobilized and stabilized by the Co−N−C interfacial structure.

As a result of the rapid coarsening kinetics of a high density of thermally activated metal atoms, heterogeneous metal catalysts of a subnanometer size and with a high metal loading have been sought for a long time, but have proven very difficult to produce [27,39]. Accordingly, we proposed that the formation of subnanometer Co clusters during GCURH should be attributed in large part to the kinetics-dominant and diffusion-constrained conditions provided by our technique. In particular, thermally activated atomic or metal-containing molecule species diffuse in the form of gas phase and grow into metal particles by collisions and combinations [20,40]. The shorter the high-temperature pulse, the lower the likelihood of atomic collisions and combinations. Based on the notion of Brownian motion in the gas phase, the motion rate (*v*) of atoms or molecules can be calculated to be on the order of centimeters per second (∼cm/s, equal to 10^7^ nm/s), which is faster than in the liquid or solid phases (Fig. 3h) [21]. During the limited μs-scale of a high-temperature pulse (*t*, 1 μs = 10^−6^ s) in GCURH, the diffusion distance (*l, l* = *v* × *t*) of reduced metal nodes of a MOF in the graphene-confined reactor is kinetically limited to the nanoscale. Therefore, the GCURH technique can be applied to synthesize MOF-derived metal clusters with high metal loading by reducing the collision and recombination of atoms (inset of Fig. 3h), and it is also applicable to other material systems such as Ni clusters (Fig. S13). In the absence of temporal and spatial constraints, the reduced metal nodes would be coarsened into nanoparticles (Fig. S14) [18,26,27,41]. Overall, the GCURH technique offers a novel and kinetically adjustable method for resolving the trade-off between subnanometer size and high metal loading (Fig. S15), which is anticipated to endow cluster catalysts with exceptionally high activity.

### Catalytic performance

**Figure 4.**
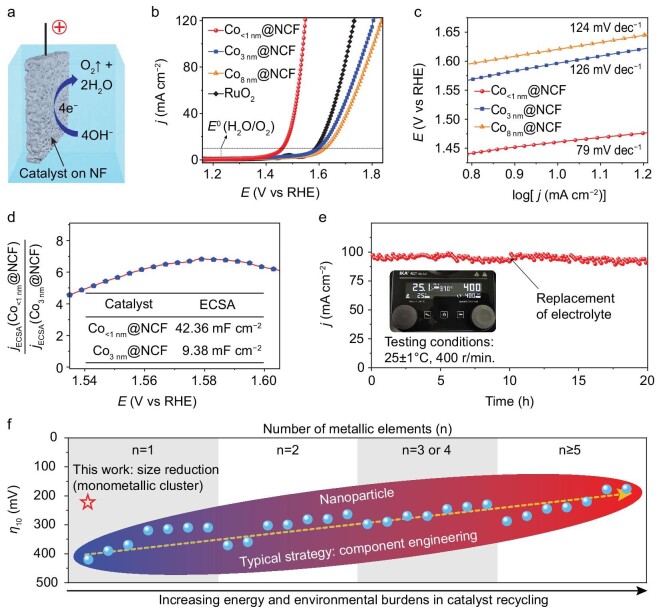
OER performance. (a) Schematic illustration of the OER in alkaline electrolyte. (b) Linear sweep voltammetry (LSV) curve measurement was conducted in Ar-saturated 1.0 M KOH at a scan rate of 5 mV s^−1^. (c) Tafel slopes. (d) *j*_ECSA_ comparison of Co_<1 nm_@NCF and Co_3 nm_@NCF, *j*_ECSA_ = *j*/ECSA. (e) Chronoamperometric curve; the data were obtained at 25 ± 1°C with a stirring speed of 400 r/min (shown in the inset). (f) Comparison of OER activity for various advanced metallic electrocatalysts. All given potentials were referenced to the reversible hydrogen electrode (RHE) through RHE calibration.

Considering the high activity of Co centers for water dissociation, electrocatalytic OER was chosen as the model reaction [11,42,43]. A Co_<1 nm_@NCF catalyst and contrast catalysts were, therefore, anchored on nickel foam and tested in an alkaline electrolyte to evaluate the performance of an as-prepared high-loading subnanometer metal cluster catalyst (Fig. 4a). In addition, the control catalysts Co_3 nm_@NCF (average Co nanoparticles are 3 nm) and Co_8 nm_@NCF (average Co nanoparticles are 8 nm) were synthesized based on our prior work [22]. Comparing the OER performance of the Co cluster and Co nanoparticle catalysts revealed size-dependent activity in the as-prepared catalysts (Fig. 4b and c). In particular, as the size of Co particles in catalysts fell from 8 nm to 3 nm, the overpotential at 10 mA cm^−2^ (*η*_10_) reduced marginally from 391 mV for Co_8 nm_@NCF to 367 mV for Co_3 nm_@NCF, both of which were inferior to that of commercial RuO_2_ (357 mV at *η*_10_). However, when the size of Co particles was reduced to subnanometer dimensions, the OER activity of Co_<1 nm_@NCF increased considerably. Accordingly, an overpotential of just 231 mV was necessary to drive a current density of 10 mA cm^−2^, outperforming RuO_2_ in terms of activity (Table S4). To understand the cause of such remarkable activity, the ECSA and the normalized current density (*j*_ECSA_ = *j*/ECSA) of Co_<1 nm_@NCF and Co_3 nm_@NCF were measured and compared (Fig. S16, Fig. 4d). The ECSA of Co_<1 nm_@NCF (42.36 mF cm^−2^) is 4.5 times greater than that of Co_3 nm_@NCF (9.38 mF cm^−2^), demonstrating that the size-decreasing strategy can effectively increase the number of active sites in Co subnanometer cluster catalysts. In addition, the *j*_ECSA_ of Co_<1 nm_@NCF is four times higher than Co_3 nm_@NCF at a potential window between 1.54 V and 1.60 V (vs. RHE), indicating that the Co center in Co_<1 nm_@NCF has a higher intrinsic activity during OER [44]. Moreover, the Co_<1 nm_@NCF catalyst maintains its stability at 100 mA cm^−2^ for 20 hours (Fig. 4e).

The OER is a vital semi-reaction in chemical production and energy conversion processes [42,43]. Accordingly, growing efforts have been made to create high-performance OER catalysts. Increasing the number of metallic elements (n) in alloy nanocatalysts (n↑ → OER↑, Fig. 4f, Table S5) is one of the effective strategies for boosting OER activity [45,46]. However, the recovery, separation and purification of each component in deteriorated catalysts would result in a significant increase in energy consumption and environmental impact due to the heterogeneous composition of the catalysts [47]. Our findings indicate that the activity of a single-component and non-noble transition metal catalyst can be greatly enhanced by reducing its size to the subnanometer scale (size↓→ OER↑, Fig. 4f), and the single metallic species would be of great significance in minimizing energy consumption and environmental pollution during catalyst recycling. In addition, the size-decreasing strategy has been reported to be effective in increasing the activity of catalysts in various material systems (e.g. oxides), but at the expense of loading [48−50]. Our GCURH technique possesses ultrafast kinetic and extreme thermodynamic conditions, hence providing a viable and novel method for resolving the trade-off between subnanometer size and high loading, which is crucial for the development of sophisticated heterogeneous catalysts. We would like to mention that although scaled-up production of metal cluster catalysts remains a formidable obstacle, the technology and the underlying mechanism suggested herein are easily automatable and transformable into a continuous production process (Fig. S17).

## CONCLUSION

Based on the unique physicochemical properties of graphene, we established, for the first time, a μs-scale GCURH technique (heating and cooling rates, ∼10^9^°C/s; peak temperature, >2000°C) for the controlled synthesis of high-loading metal cluster catalysts. First, by irradiating sufficient laser energy, the high absorption and low specific heat capacity endow the optically thin graphene sheets with an ultrafast heating rate. Second, the high thermal emissivity and low specific heat capacity of laser-irradiated graphene endow it with an extraordinarily fast radiative cooling rate. Third, the graphene's intrinsic properties of high thermal stability, low permeability and high flexibility allow it to function as an interlayer confined nanoreactor for high-temperature processes. By pyrolyzing ZIF-67, the GCURH technique is, therefore, capable of kinetically restricting the diffusion distance of metal atoms and synthesizing subnanometer Co cluster catalysts with Co loading of up to 27.1 wt% or 7.3 at%, featuring one of the highest size-loading combinations to date. Electrocatalytic OER tests show that the Co cluster catalyst presents exceptional activity and durable stability during 20 hours of an OER reaction. At a current density of 10 mA cm^−2^, the overpotential is decreased to 231 mV, which is superior to the activity of commercial RuO_2_ or Co nanocatalysts, and comparable to that of sophisticated multicomponent noble metal catalysts. Moreover, the single metal component of the as-prepared Co cluster catalyst reduces its energy consumption and environmental impact during recycling. Therefore, the as-developed GCURH method provides a novel and promising approach for exploring sophisticated and sustainable subnanometer cluster catalysts for clean energy utilization and fine chemicals synthesis.

## MATERIALS AND METHODS

Detailed materials and methods are available in the Supplementary Data.

## Supplementary Material

nwad081_Supplemental_File
