# Supplementary material for: Graphene-confined ultrafast radiant heating for high-loading subnanometer metal cluster catalysts
Source: Natl Sci Rev. 2023 Mar 23;10(6):nwad081. doi: 10.1093/nsr/nwad081 (PMC10317146; doi:10.1093/nsr/nwad081)
Supplement: nwad081_Supplemental_File [file nwad081_supplemental_file.docx]

**Supporting Information**

**Graphene-confined ultrafast radiant heating for high-loading subnanometer metal cluster catalysts**

Ye-Chuang Han,^1,2,†^ Jun Yi,^1,2,†^ Beibei Pang,^3,†^ Ning Wang,^4^ Xu-Cheng Li,^1^ Tao Yao,^3^ Kostya S. Novoselov,^1,5,*^ and Zhong-Qun Tian.^1,2*^

^1^State Key Laboratory of Physical Chemistry of Solid Surfaces, College of Chemistry and Chemical Engineering, Graphene Industry and Engineering Research Institute, Xiamen University, Xiamen 361005, China.

^2^Innovation Laboratory for Sciences and Technologies of Energy Materials of Fujian Province (IKKEM), Xiamen 361005, China.

^3^National Synchrotron Radiation Laboratory, University of Science and Technology of China, Hefei 230029, China.

^4^Faculty of Environment and Life, Beijing University of Technology, Beijing 100124, China.

^5^Institute for Functional Intelligent Materials, National University of Singapore, Singapore 117544, Singapore.

^†^These authors contributed equally to this work.

*Corresponding author.

**Email**: [zqtian@xmu.edu.cn](mailto:zqtian@xmu.edu.cn) (Z. T). [kostya@nus.edu.sg](mailto:kostya@nus.edu.sg) (K. N).

**Materials and Methods**

***Synthesis of ZIF-67****.* ZIF-67 was synthesized as reported by Chong et al [1].Co(NO_3_)_2_·6H_2_O (1.0 g) was dissolved in 50 mL of methanol using ultrasonification, followed by the addition of a methanolic solution (80 mL) of 2-methylimidazole (MeIM, 2.5 g). Subsequently, the mixtures were vigorously agitated at 800 r/min for 30 min at room temperature to form a heterogeneous violet mixture, which was sealed and stored at room temperature for 12 h. The violet crystals were separated by centrifugation (12000 rpm) and washed thrice thrice in methanol and twice in deionized water. ZIF-67 was finally freeze-dried for 24 h (−58 °C, 5~8 Pa).

***Synthesis of Co_<1 nm_@NCF***. The confinement of MOF crystals between GO interlayers can be achieved by a simple liquid mixing and then a drop-cast procedure. First, monolayer GO with lateral size of 3 μm to 5 μm was purchased from Hangzhou Gaoxi Technology Co., Ltd. (GX-GO-1), and the MOF crystals (ZIF-67) with a particle size distribution of 200 nm to 300 nm were synthesized based on our previous work [2]; the remarkably smaller ZIF-67 crystals can be wrapped in GO interlayers. Second, 6 mg of ZIF-67 powder was ultrasonically dispersed in 2 mL of deionized water that contained 3 mg of GO for 2 hours, and formed a uniform mixture of ink; then, 10 μL of as-prepared ink was drop-casted onto the quartz glass and dried at ambient temperature, respectively. Third, the dried slurry was tightly covered by another piece of quartz glass to prevent the sample from splashing during the laser shock, and the whole sample was further dried for 6 hours in an oven at 80°C. Finally, one pulse of a laser aimed at the targeted sample location was triggered (wavelength, 355 nm; duration, 6 ns; InnoL as SpitLight600, PulsePower Inc.).

***Preparation of working electrode***. The as-prepared catalysts (2.0 mg) were dispersed ultrasonically in 490 μL of isopropanol containing 10 μL of Nafion solution (Alfa Aesar). After 30 min of ultrasonication, 250 μL of homogenous ink was drop-cast onto 1.0 cm^2^ of nickel foam and allowed to dry at room temperature. Nickel foam-supported catalysts were used as the working electrodes, respectively.

***Electrochemical measurements***. Using a CHI660E electrochemical workstation and a standard three-electrode cell in an Ar-saturated 1.0 M KOH electrolyte, electrochemical experiments were conducted. Pt wire and Ag/AgCl were employed as the reference and counter electrodes, respectively. For the OER measurement, a scanning rate of 5 mV s^−1^ was applied to obtain LSV curves. Herein, all given potentials were referenced to the RHE by RHE calibration according to the equation $E\left( \mathrm{RHE} \right)=E(Ag/AgCl)+0.197+0.0591\times pH$, where the pH value of the 1.0 M KOH aqueous solution is 14. All polarization curves presented herein were adjusted with 80% iR correction.

***Material characterization***. The phase structure and crystallinity of the as-prepared catalysts were determined by using XRD on a Rigaku Ultima-IV system with Cu Kα radiation (λ = 1.5406 Å). XPS measurements of the as-synthesized catalyst were obtained using an Escalab 250Xi system, and the spectra were collected with a monochromatic Al Kα source. The morphologies, HAADF–STEM, EDX elemental mapping analyses, and microstructures of the prepared catalysts were determined using an FEI Tecnai TF20 system that operated at an accelerating voltage of 200 kV. The spherical aberration-corrected HAADF–STEM image of prepared subnanocobalt clusters was captured using a Titan Themis Z microscope operating at 300 kV accelerating voltage (collecting angles, 22~135 mrad). For XANES and EXAFS measurements, 6 mg of sample was homogeneously mixed with 34 mg of graphite and then pressed into circular pellets with a diameter of 8 mm for subsequent X-ray absorption fine structure (EXAFS) measurements under ambient conditions. The Co K-edge (7709 eV) EXAFS spectra were obtained at the BL14W1 station of the Shanghai Synchrotron Radiation Facility (SSRF), China. The storage rings of the SSRF were operated at 3.5 GeV with a maximum current of 210 mA.

**Pulsed laser heating and radiative cooling of graphene oxides (GO):** Upon illumination, the electrons and phonons of GO are excited to nonequilibrium states, and spontaneously release the excess energy to decay to equilibrium states [3]. The released energy can convert to disordered internal energy and result in photo-induced heating. The pulse duration time is essential to determine the photothermal effect of materials under pulse illumination. Generally, within the first picosecond after excitation, the absorption of ultrafast pulse leads to a nonequilibrium population of electrons, where electrons are heated to a transient high temperature, while the lattice temperature remains unchanged. The excited electrons and holes subsequently decay to the band edge via emitting phonons, such interplays between electrons and phonons eventually results in heat generation via energy dissipation to the random lattice vibrations. Besides, the non-radiative recombination between electrons and holes also contribute to heat generation, while the timescale of ~ ns or μs is much smaller than the electron-phonon interaction. Since the GO is a direct band gap material, where radiative recombination dominates, the non-radiative thermal contribution is negligible and the major photothermal effect origins from the electrons/holes-phonons interactions. Additionally, at a nanosecond scale, the electrons and lattice have reached a thermal equilibrium. Therefore, the temperature distribution of GO can be described by a classical heat transfer equation as:

$$\rho c\frac{\partial T}{\partial t}+\rho c\mathbf{u}\nabla T+\nabla\cdot\left( \mathbf{q} \right)=Q_{0} (1)$$

where ρ is mass density, c is heat capacity, k is thermal conductivity, T is the time-dependent temperature of GO, $\mathbf{u}$ is the velocity, and $\mathbf{q}$ is the density of heat flux, respectively. The local heat source Q_0_ origin from the optical absorption of GO. The first term in the left bracket of equation 1 describes the temperature increase due to thermal accumulation, the second term describes the convection heat transfer, and the third term describes the heat flux which includes heat conduction, convection, and thermal radiation as:

$$\mathbf{q}=-k\nabla T+\boldsymbol{q}_{s} \left( 2 \right)$$

$$\boldsymbol{n}\cdot\boldsymbol{q}_{s}=-\varepsilon\sigma\left( T^{4}-T_{0}^{4} \right)-h\left( T-T_{0} \right) (3)$$

where $k$ is the thermal conductivity, $\varepsilon$ is the surface emissivity, $\sigma$ is the Stefan-Boltzmann constant, *h* is the convection coefficient, and T_0_ is the ambient temperature, respectively. Therefore, for the system with high temperature and low thermal conductivity, the thermal radiations dominate the thermal transfer process.

To evaluate the temperature distribution, we begin with calculating the optical absorption of GOs to obtain the joule heating terms Q_0_. The frequency-dependent refractive constant of GO was described by a sum of Lorentzian oscillators as [4]:

$$\varepsilon_{\omega}=\varepsilon_{\infty}+\sum_{i} \frac{f_{i}}{\omega_{i}^{2}-\omega^{2}-i\gamma_{i}\omega} (4)$$

Additionally, it should be noted that the detailed parameters were obtained by fitting the measured UV-Vis spectra of GO flake in solution, the fitted frequency-dependent n and k are shown in Supplementary Fig. 5.


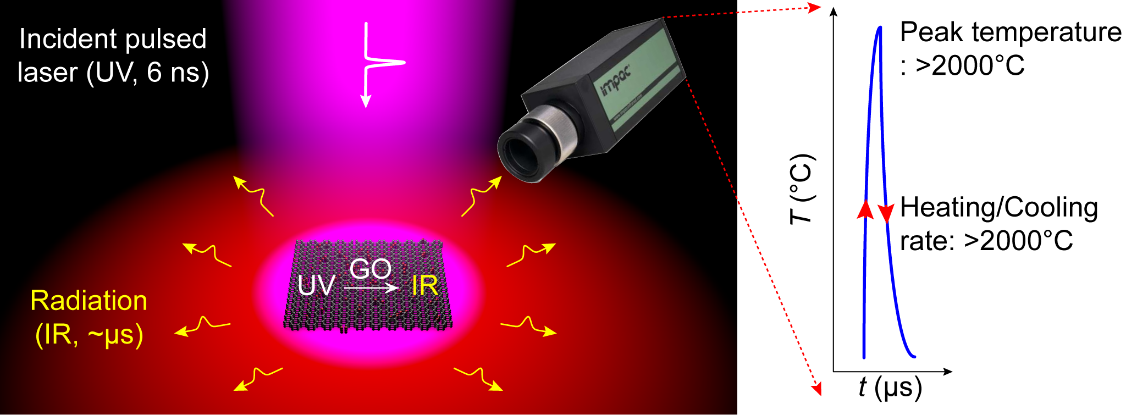


**Figure S1.** Schematic illustration of the laser-triggered and GO-mediated GCURH method, IR pyrometer equipped with a high-speed data-acquisition workstation was used for temperature measurement with a sampling rate of one temperature point per microsecond (μs).


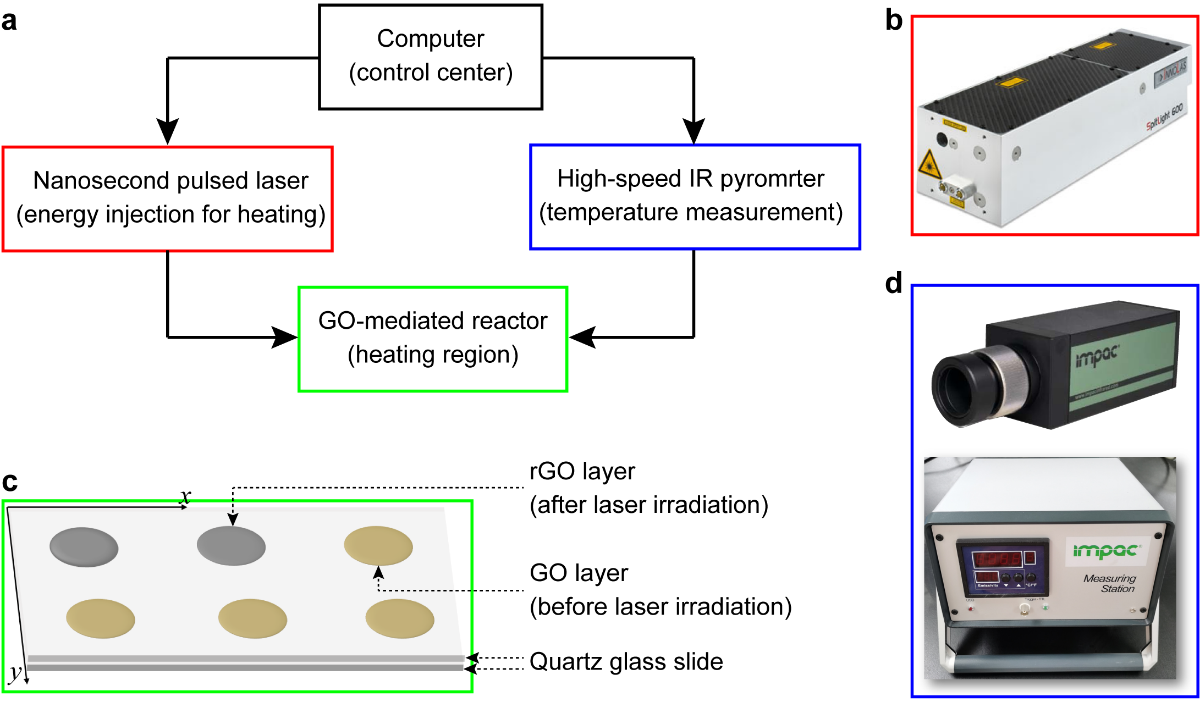


**Figure S2.** The ns laser-triggered GCURH system. (a-d) Schematic illustration and corresponding experimental setup of the radiant heating system.


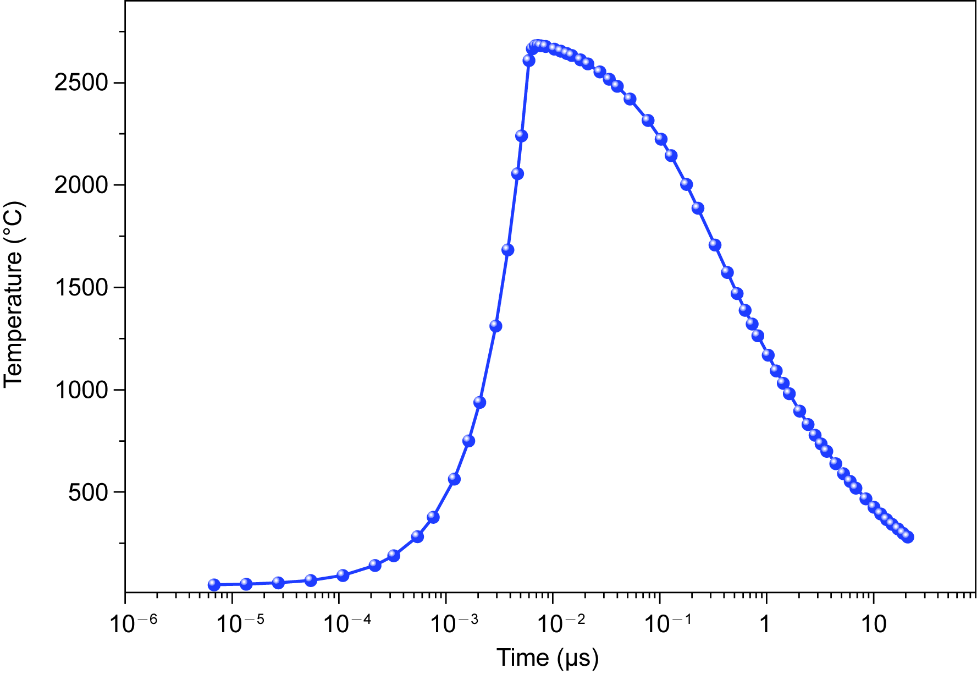


**Figure S3.** The calculated (red dots) averaged temperature of GO cavity as a function of time.

**Note**: The time-dependent temperature variation was evaluated by equation (1-3) in the same model geometry. Fig. S3 shows the calculated average temperature as a function of time. The temperature raises from room temperature to ~2,700°C within 10 ns was observed, and gradually cool down to below ~700°C in 3.6 $\mu$s. The results well reproduce the experimental observation of ~ 10^9^ °C/s cooling rate, additionally, the temperature decrease can be perfectly fitted by T^4^ law, which confirms a dominated radiative heat transfer process during the photothermal effect.


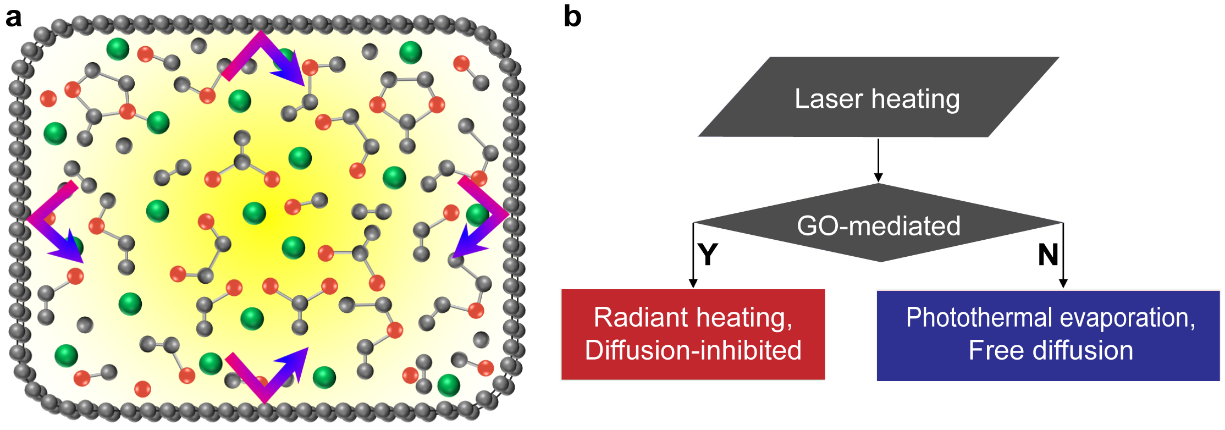


**Figure S4.** The role of GO in the construction of the diffusion-inhibited high-temperature reactor. (a) The inhibition of thermally activated atomic or molecular diffusion. (b) The mechanism comparison between GO-mediated laser heating and direct laser heating.


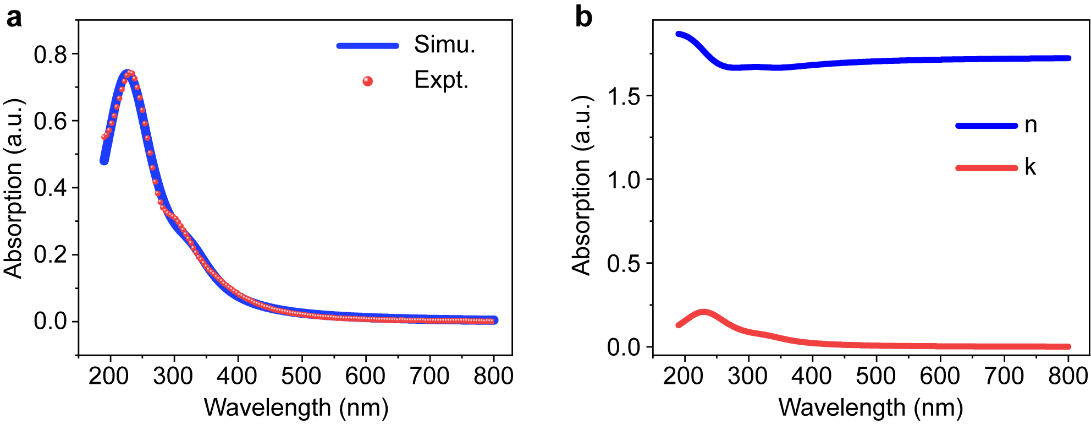


**Figure S5.** (a) The measured (red dots) and calculated (blue line) absorption spectra of GO flake in solution. (b) The Lorentzian model fitting of the refractive index of GO flake.


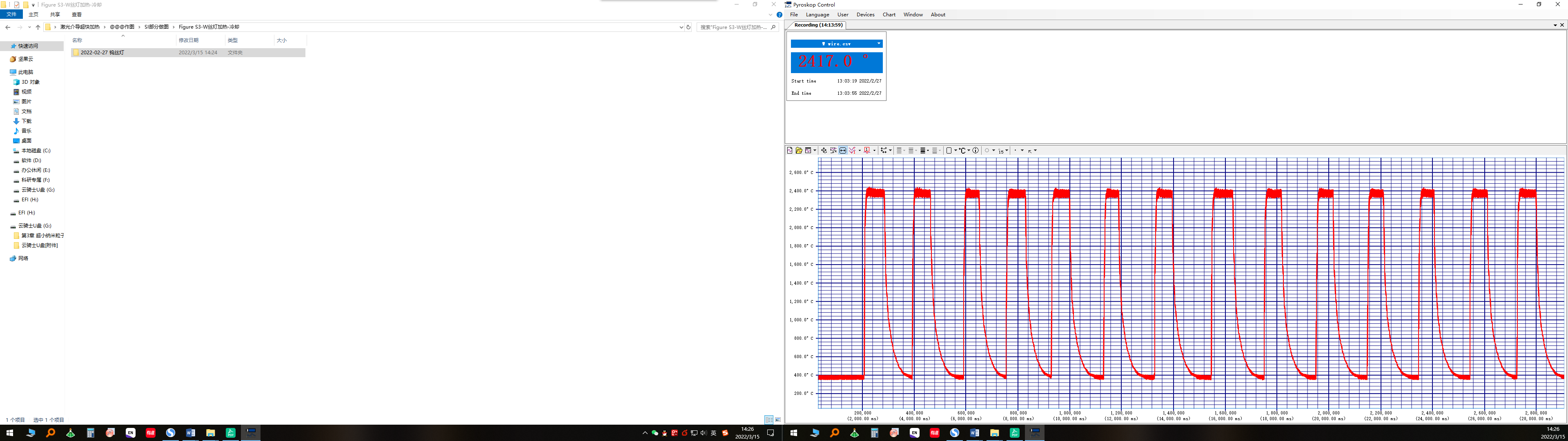


**Figure S6.** Temperature variation during switch on (heating, temperature rise) and switch off (cooling, temperature down) of the tungsten lamp.

**Figure S7.** The number of publications about “MOF pyrolysis” or “MOF-derived” materials, showing the flourishing of this field.


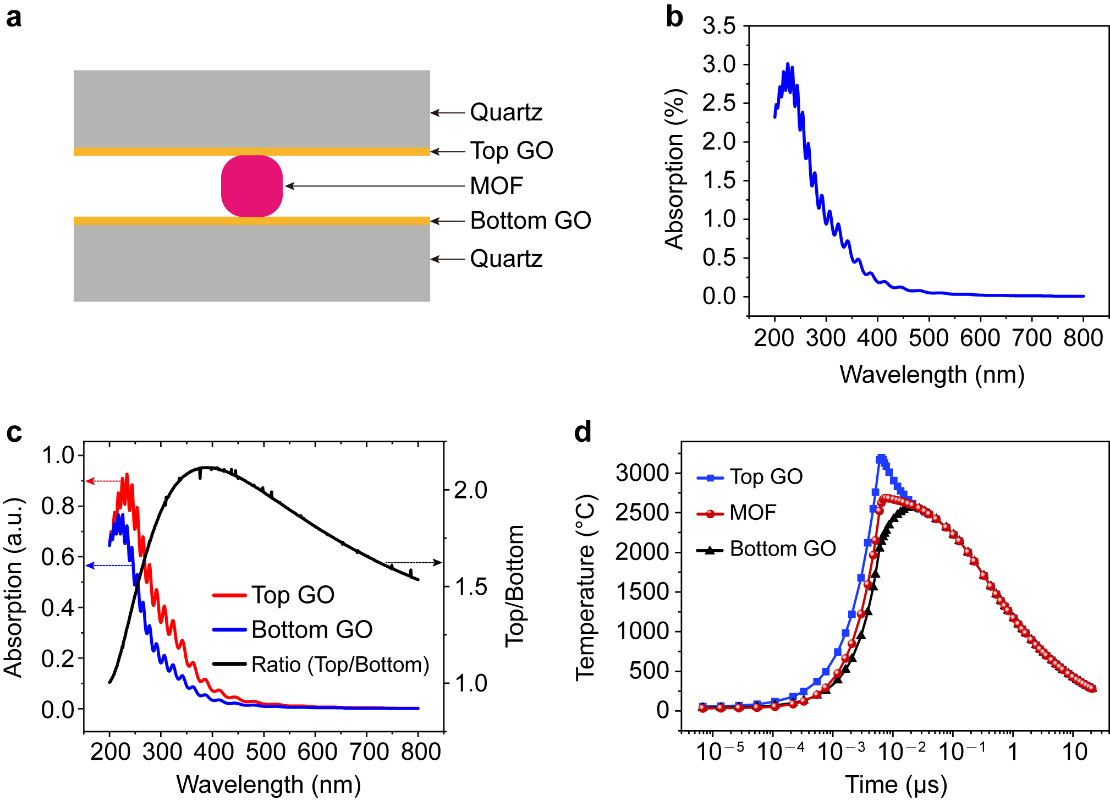


**Figure S8.** (a) Schematic illustration of the sample. (b) The calculated absorption spectrum of quartz capsulated GO cavity. (c) The calculated absorption by top GO layer and bottom GO layer, and the absorption ratio between top and bottom layer, respectively. (d) The calculated temperature of the top GO layer, the bottom GO layer, and the MOF, respectively.

**Note**: We further simulate the optical absorption of GO in the experimental configuration, where a GO-MOF-GO sandwich was encapsulated by a pair of quartz (Fig. S8a). The MOF particle is considered transparent in the visible range, and the refractive index of quartz and MOF is 1.45 and 1.1, respectively. Fig. S8b shows the calculated optical absorption of the encapsulated GO-MOF-GO sample, which exhibits a peak absorption of ~ 3.0% around 225 nm, and the relative absorption from the top GO layer and the bottom GO layer is shown in Fig. S8c The slight difference in absorption could lead to a non-uniform temperature distribution between the top and the bottom layer.

The time-dependent temperature was evaluated by equation (1-3) in the same model geometry. Fig. S8d shows the calculated average temperature as a function of time. The temperature rises from ambient temperature to ~3,000°C within 10 ns was observed, and gradually cools down to below 1,000°C in 3.6 $\mu$s. The results well reproduce the experimental observation of ~ 10^9^ °C/s cooling rate, additionally, the temperature decrease can be perfectly fitted by the T^4^ law, which confirms a dominated radiative heat transfer process during the photothermal effect. Due to the direct thermal contact between GO and the sandwiched MOF, the heat flux can be efficiently injected from heated GO to the MOF. Supplementary Fig. 8d shows the detailed temperature distribution of the Top GO, the Bottom GO, and the MOF, respectively. It shows that the thermal equilibrium has been established in 15 ns after the pulse illumination. We note that in the simulation, the near-field radiative heat transfer between top and bottom GO is not included, which can further increase the thermal exchange between GO layers via near-field coupling. This simplification holds because the concerned time scale of simulation and experiment is in $\mu$s scale, where the thermal equilibrium has already been reached. Besides, the chemical changes such as GO reduction, and reaction heats are not included in the simulation, due to the complexity.


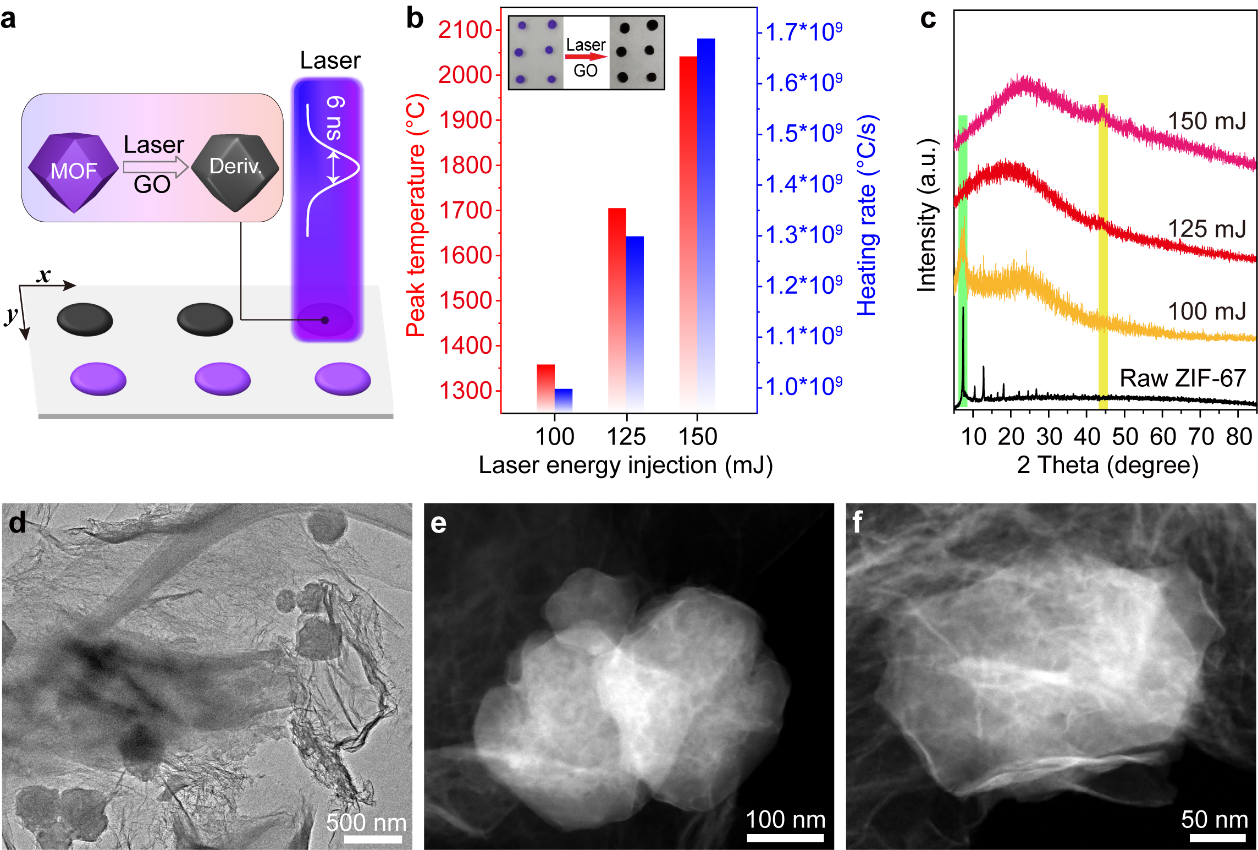


**Figure S9.** Graphene-confined radiant pyrolysis of ZIF-67. (a) Schematic illustration of the preparation process. (b) Peak temperature and heating rate by different laser energy injections. (c) XRD patterns of ZIF-67 derivatives were prepared with different laser energy injections. (d) TEM image and (e, f) HAADF-STEM images of ZIF-67 derivatives prepared by the GCURH method with 125 mJ of laser energy injection.


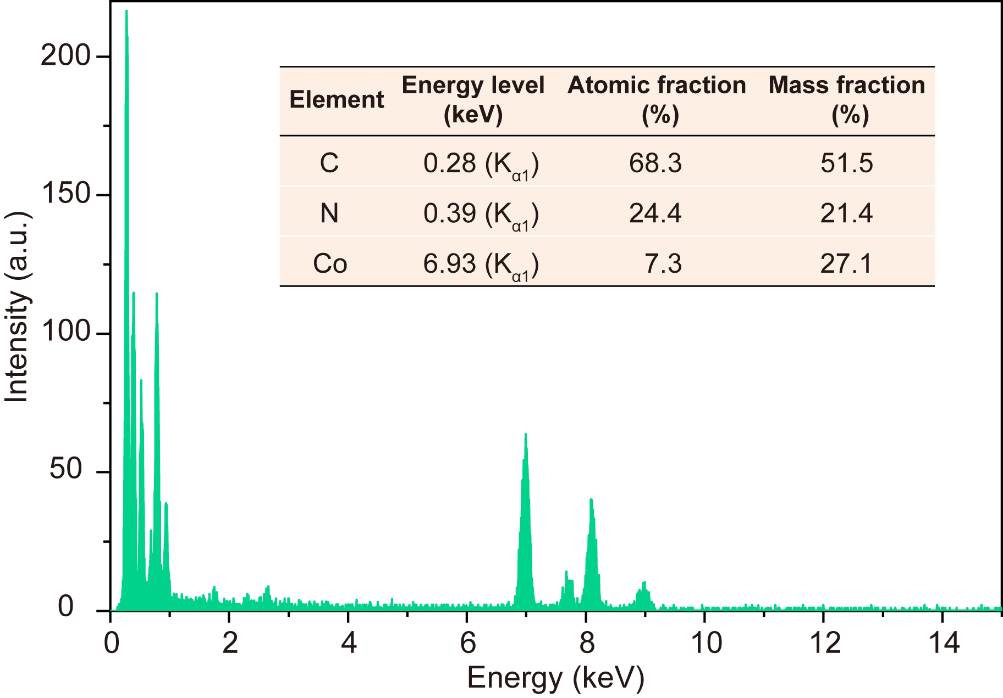


**Figure S10.** EDX spectra analysis of ZIF-67 derivatives after single-pulse of GCURH treatment (wavelength, 355 nm; pulse duration, 6 ns; irradiated laser energy, 125 mJ).


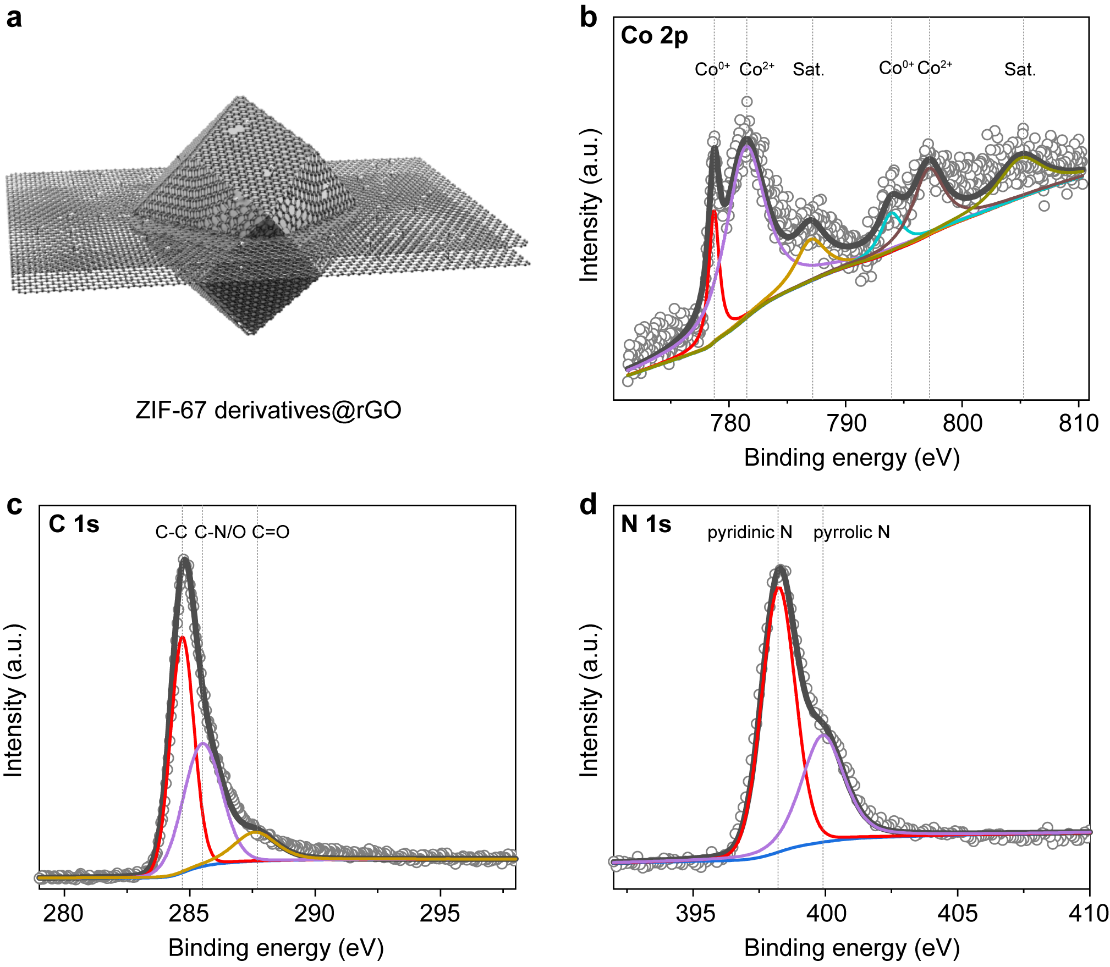


**Figure S11.** XPS characterization of ZIF-67 derivatives@rGO. (a) Schematic illustration of ZIF-67 derivatives@rGO. (b) High-resolution Co 2p XPS spectra. (c) High-resolution C 1s XPS spectra. (d) High-resolution N 1s XPS spectra.


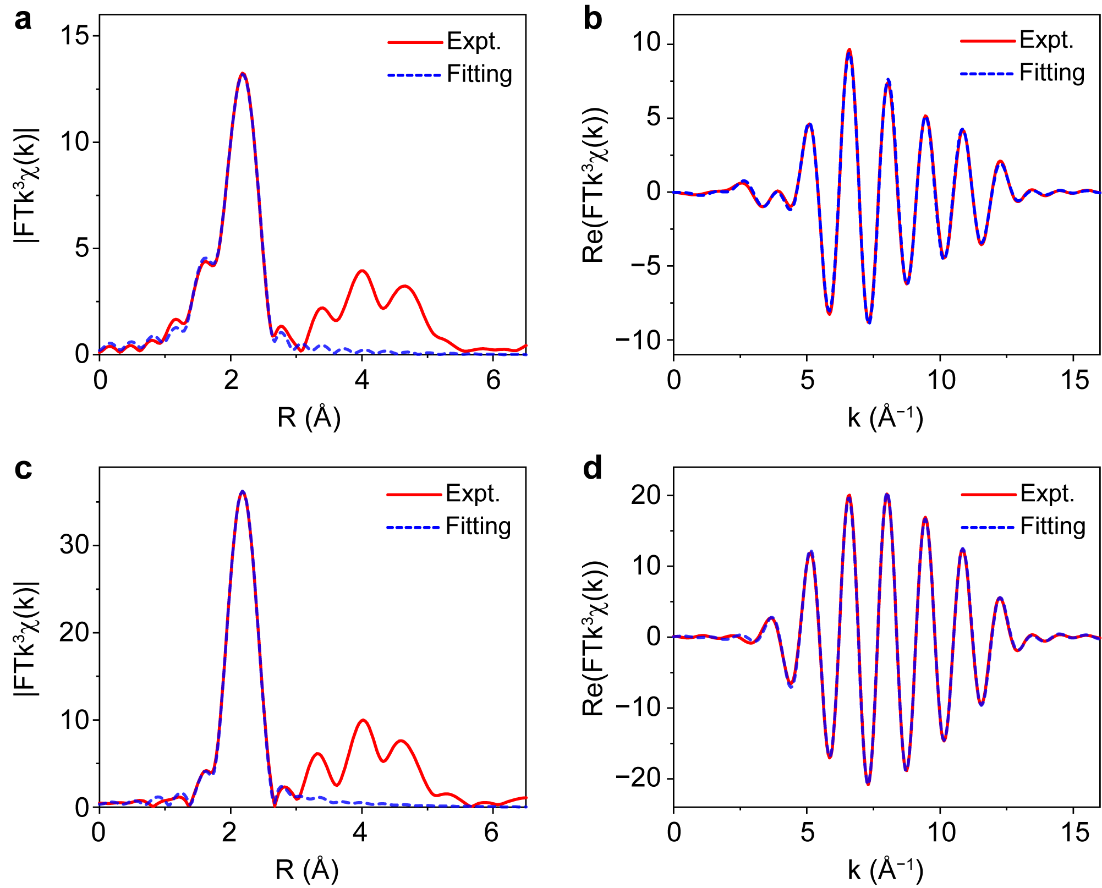


**Figure S12.** (a) Least-squares curve-fitting analysis of EXAFS spectra at the Co *K*-edge, and (b) Corresponding Re(k^3^χ(*k*)) oscillations of Co_<1 nm_@NCF. (c) Least-squares curve-fitting analysis of EXAFS spectra at the Co *K*-edge, and (d) Corresponding Re(k^3^χ(*k*)) oscillations of Co foil.


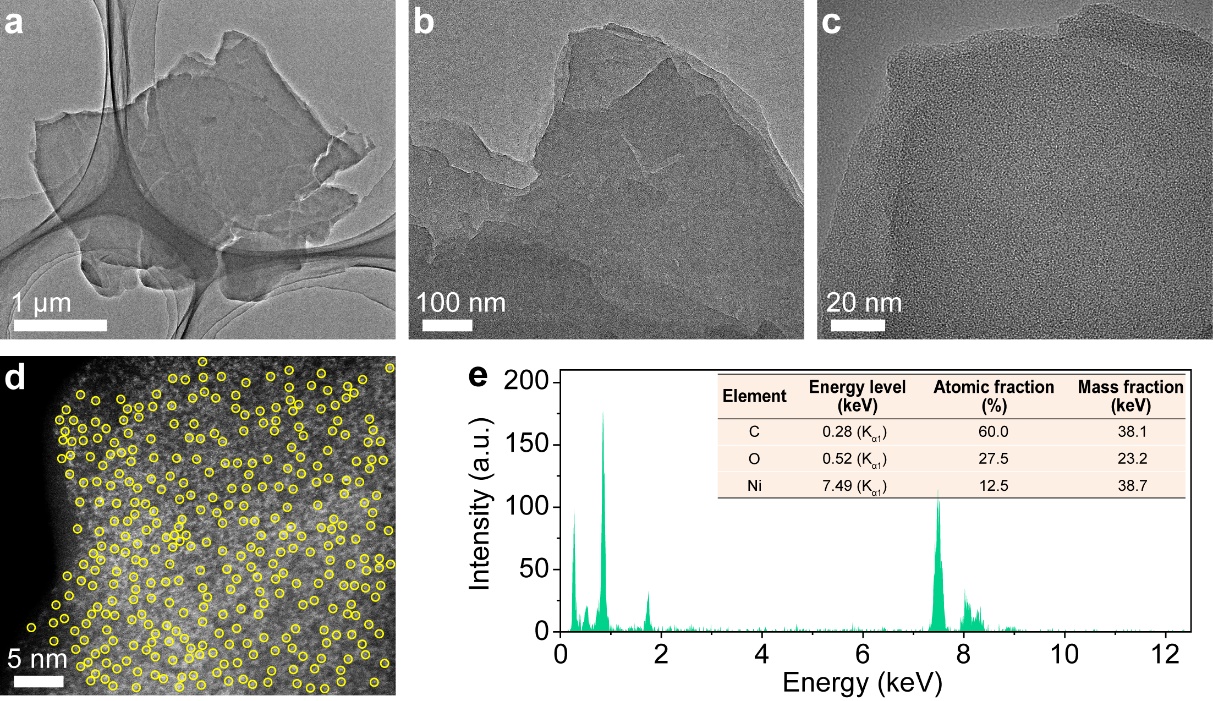


**Figure S13.** Characterization of MOF-derived Ni clusters. (a-c) TEM images of carbon supported Ni clusters that were prepared by GCURH treatment of Ni-based MOF. (d) Spherical aberration-corrected HAADF-STEM image of densely dispersed Ni clusters on the carbon support. (e) EDX spectra analysis of Ni-based MOF derivatives after single-pulse of GCURH treatment (wavelength, 355 nm; pulse duration, 6 ns; injected laser energy, 125 mJ).

**Note:** Ni-based MOF (Ni-TBAPy) was synthesized based on our prior work [2].


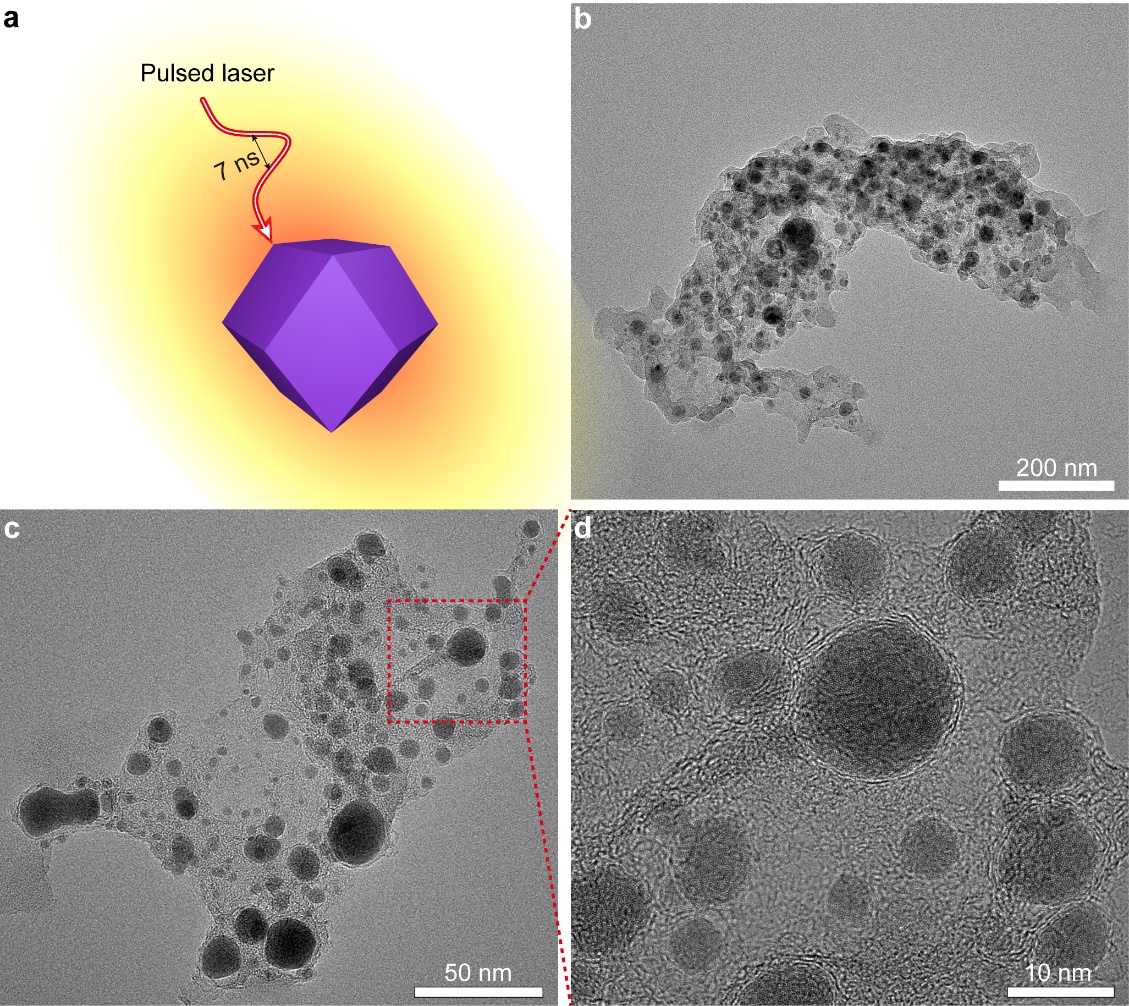


**Figure S14.** ZIF-67 derivatives were prepared by one pulse of direct laser heating. (a) Schematic illustration. (b-d) TEM images**.** The wavelength, 1,064 nm; pulse duration, 7 ns; pulse number, 1 pulse; laser energy injection, 125 mJ.

**
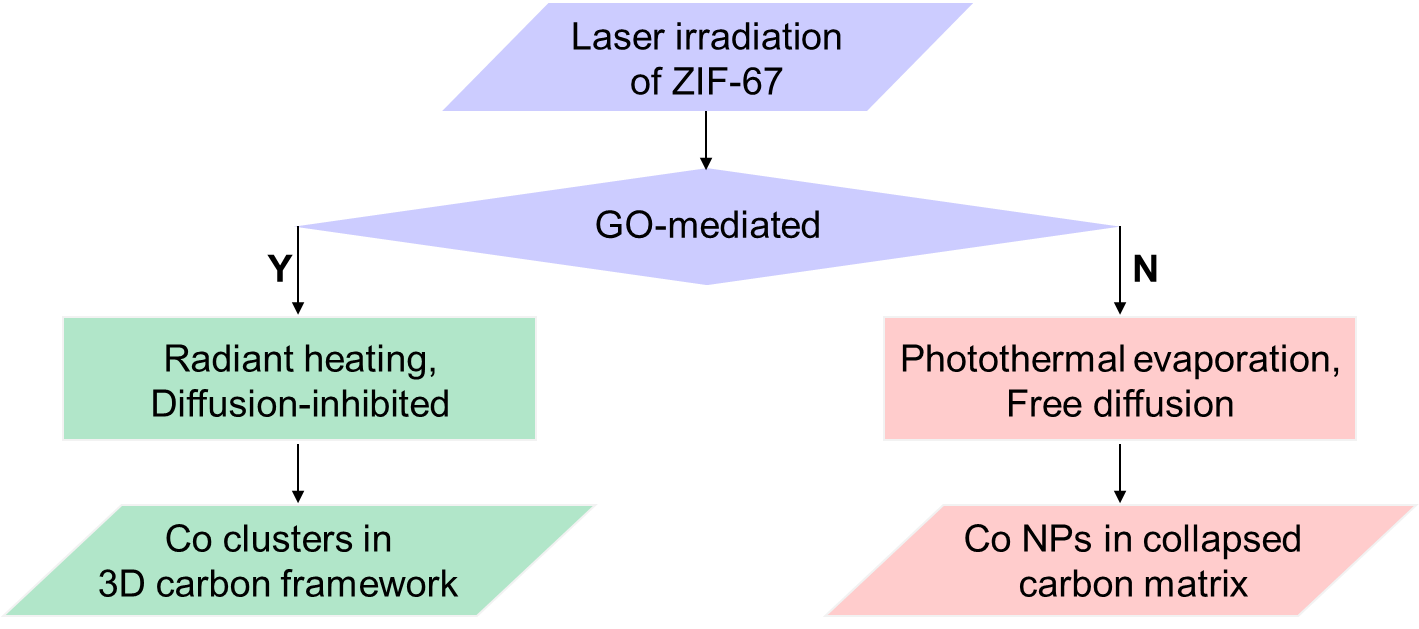
**

**Figure S15.** Comparison of GO-mediated and laser-triggered radiant heating and typical laser heating by direct laser irradiation.


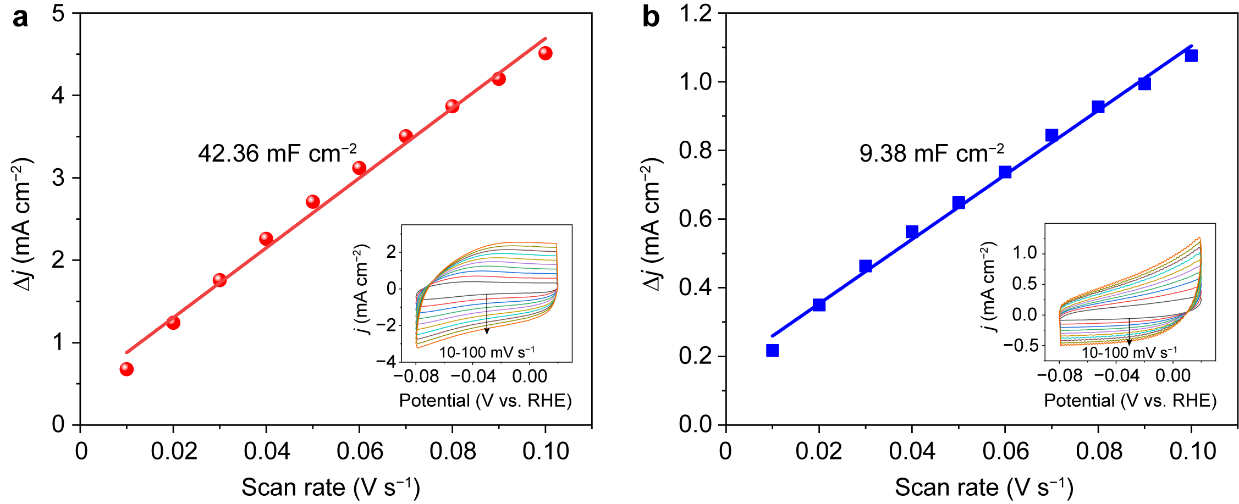


**Figure S16.** Double-layer capacitance (C_dl_) was used to estimate the ECSA of the catalysts. (a) Co_<1 nm_@NCF, (b) Co_3 nm_@NCF; inset are the cyclic voltammetry (CV) curves obtained at different scan rates.


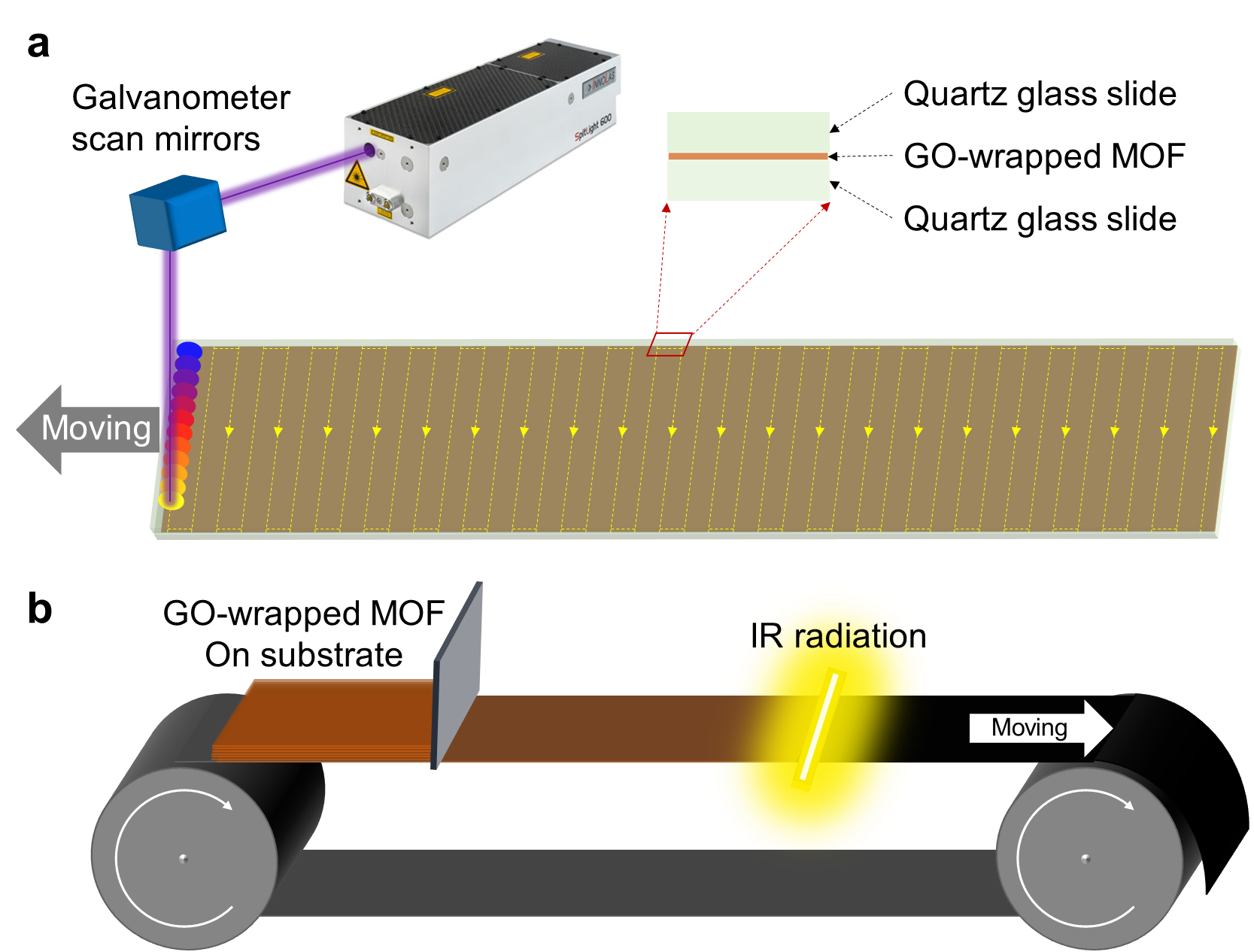


**Figure S17.** (a) Schematic illustration for the scale-up synthesis of metal cluster catalysts. GO wrapped MOF was pressed into a thin film between two quartz glass slides; a pulsed laser equipped with galvanometer scan mirrors was applied to ultrafast radiant heating of GO wrapped MOF for synthesizing metal cluster catalysts, and the yellow dashed line was the programmed target path for laser scan. (b) Schematic illustration for scaled-up production of single atoms using non-contact radiative heating in an automated, streamlined process; this design has referred to Yao’s work [5].

**Table S1.** Comparison of the heating rate and total heating duration in the various pulsed heating methods.

| **Method** | **Heating rate (°C/s)** | **Duration**  **(heating and cooling)** | **Reference** |
| --- | --- | --- | --- |
| GCURH | 10^9 | ~25 μs | This work |
| Direct laser heating | 6*10^5 | 14 ms | [6] |
| Direct laser heating | 10^4 | 50 ms | [7] |
| Joule heating | 4*10^5 | 5 ms | [8] |
| Joule heating | 10^5 | 55 ms | [9] |
| Joule heating | 2*10^4 | 100 ms | [10] |
| Joule heating | 3*10^5 | 110 ms | [11] |
| Joule heating | 10^5 | ~60 ms | [12] |
| Joule heating | 3.6*10^5 | 55 ms | [13] |
| Joule heating | 10^4 | 75 ms | [14] |
| Joule heating | 2.7*10^4 | 100 ms | [5] |
| Joule heating | 10^3 | 5 s | [15] |
| Joule heating | 8.75 | 3 min | [16] |
| Microwave heating | 2.5*10^4 | 400 ms | [17] |
| Microwave heating | 550 | 13 s | [18] |
| Microwave heating | 100 | 15 s | [19] |
| Microwave heating | 160 | ~10 s | [20] |
| Induction heating | 230 | 16 min | [21] |
| Induction heating | 8.6 | ~2.5 min | [22] |
| Induction heating | 3.3 | 60 min | [23] |
| Infrared heating | 1~30 | ~h | [24 |
| Tube furnace heating | <1 | ~h | [2] |

**Table S2.** Comparison of the metal loading in cluster catalysts.

| **Catalyst** | **Weight percent**  **(wt%)** | **Atomic percent**  **(at.%)** | **Reference** |
| --- | --- | --- | --- |
| Co_<1 nm_@NCF | 27.1 | 7.3 | This work |
| Cu clusters/DRC | ~5 | NG | [25] |
| Co_SC_-N-C | 3.15 | NG | [26] |
| Fe_2_-N-C | 0.38 | NG | [27] |
| Fe_3_-N-C | 0.39 |  |  |
| Pt@MCM-22 | 0.17 | NG | [28] |
|  | 0.3 |  |  |
| Ru/NC | 0.4 | NG | [29] |
| Pt@Zeolite | 0.4 | NG | [30] |
| Au/MWCNT | 0.1 | NG | [31] |

NG: Not given in the literature.

**Table S3.** Structural parameters extracted from quantitative EXAFS curve-fitting using the ARTEMIS module of IFEFFIT.

| Sample | Path | CN | R (Å) | σ^2^ (10^−3^Å^2^) | | ΔE_0_ (eV) | R-factor |
| --- | --- | --- | --- | --- | --- | --- | --- |
| Co foil | Co-Co | 12 | 2.49 | 6.1 | 7.5 | | 0.001 |
| Co_<1 nm_@NCF | Co-N | 2.6 | 1.98 | 7.2 | 8.0 | | 0.002 |
|  | Co-Co | 5.5 | 2.49 | 7.5 | 7.6 | |  |

CNs, coordination numbers; R, bonding distance; σ^2^, Debye-Waller factor; ΔE_0_, inner potential shift.

**Table S4.** The catalytic activity of commercial RuO_2_ catalysts in OER tests.

| Catalyst | Electrolyte | Scan rate (mV s^−1^) | η_10_ (mV) in OER tests | Reference |
| --- | --- | --- | --- | --- |
| RuO_2_ | 1.0 M KOH | 5 | 357 (80% iR-compensation) | This work |
| RuO_2_ | 1.0 M KOH | 2 | 367 (95% iR-compensation) | [32] |
| RuO_2_ | 0.1 M KOH | 5 | >400 | [33] |
| RuO_2_ | 1.0 M KOH | 10 | ~354 | [34] |
| RuO_2_ | 1.0 M KOH | 5 | 367 (95% iR-compensation) | [35] |
| RuO_2_ | 0.1 M KOH | 5 | 530 | [36] |
| RuO_2_ | 1.0 M KOH | 5 | 350 (with iR-compensation) | [37] |
| RuO_2_ | 1.0 M KOH | 5 | 332 (95% iR-compensation) | [38] |
| RuO_2_ | 0.1 M KOH | 5 | 328 | [39] |
| RuO_2_ | 0.1 M KOH | 10 | 370 (with iR-compensation) | [40] |

**Table S5.** OER activity of heterogeneous metal catalysts in alkaline electrolyte.

| **Catalyst** | **Number of metallic**  **Elements (n)** | ***η*_10_**  **(mV)** | **Reference** |
| --- | --- | --- | --- |
| Co_<1 nm_@NCF | 1 | 231 | This work |
| Co@NC | 1 | 420 | [41] |
| Ni@NiO/N–C | 1 | 390 | [42] |
| Co@NC | 1 | 370 | [43] |
| Co/CNFs | 1 | 320 | [44] |
| P-Co@NC | 1 | 315 | [45] |
| Co/N-CNT | 1 | 310 | [46] |
| Ni-NG | 1 | 310 | [47] |
| Co_3_Fe_7_@Fe_2_N | 2 | 371 | [48] |
| m-NiFe/CN_x_ | 2 | 360 | [49] |
| Co_0.75_Fe_0.25_@NC | 2 | 303 | [50] |
| FeNi@N-CNT | 2 | 300 | [51] |
| NiFe@C | 2 | 281 | [52] |
| FeNi@Graphene | 2 | 280 | [53] |
| Co_3_Fe_7_@NCNTFs | 2 | 264 | [54] |
| NiCoFe-G | 3 | 298 | [55] |
| PtFeNi | 3 | 290 | [56] |
| FeCoNi | 3 | 246 | [57] |
| NiCo/Fe_3_O_4_/MOF-74 | 3 | 238 | [58] |
| FeCoNi@N-GT | 3 | 230 | [59] |
| FeCoNiMn@NC | 4 | 270 | [60] |
| FeCoNiCu | 4 | 270 | [61] |
| FeCoNiCrNb | 5 | 288 | [62] |
| AlCrFeNiCu | 5 | 270 | [63] |
| AlNiCoRuMo | 5 | 245 | [64] |
| AlFeCoNiCr | 5 | 240 | [65] |
| CoCrFeNiMo | 5 | 220 | [66] |
| PtAuPdFeNi | 5 | 178 | [67] |
| PtIrCuCrNi | 5 | 176 |  |

**Reference**

1. Chong L, Wen J, Kubal J*, et al.* Ultralow-loading platinum-cobalt fuel cell catalysts derived from imidazolate frameworks. *Science* 2018; **362**: 1276-81.

2. Han Y-C, Liu M-L, Sun L*, et al.* A general strategy for overcoming the trade-off between ultrasmall size and high loading of MOF-derived metal nanoparticles by millisecond pyrolysis. *Nano Energy* 2022; **97**: 107125.

3. Chen G. *Nanoscale energy transport and conversion : a parallel treatment of electrons, molecules, phonons, and photons*: Oxford ; New York: Oxford University Press, 2005.

4. Kravets VG, Marshall OP, Nair RR*, et al.* Engineering optical properties of a graphene oxide metamaterial assembled in microfluidic channels. *Opt Express* 2015; **23**: 1265-75.

5. Yao Y, Huang Z, Xie P*, et al.* High temperature shockwave stabilized single atoms. *Nat Nanotechnol* 2019; **14**: 851-7.

6. Wu Y, Huang Z, Jiang H*, et al.* Facile synthesis of uniform metal carbide nanoparticles from metal-organic frameworks by laser metallurgy. *ACS Appl Mater Interfaces* 2019; **11**: 44573-81.

7. Yang Y, Yao YG, Kline DJ*, et al.* Rapid laser pulse synthesis of supported metal nanoclusters with kinetically tunable size and surface density for electrocatalytic hydrogen evolution. *ACS Appl Nano Mater* 2020; **3**: 2959-68.

8. Yao YG, Chen FJ, Nie AM*, et al.* In situ high temperature synthesis of single -component metallic nanoparticles. *ACS Cent Sci* 2017; **3**: 294-301.

9. Yao YG, Huang ZN, Xie PF*, et al.* Carbothermal shock synthesis of high-entropy-alloy nanoparticles. *Science* 2018; **359**: 1489-94.

10. Yao YG, Fu KK, Yan CY*, et al.* Three-dimensional printable high-temperature and high-rate heaters. *ACS Nano* 2016; **10**: 5272-9.

11. Luong DX, Bets KV, Algozeeb WA*, et al.* Gram-scale bottom-up flash graphene synthesis. *Nature* 2020; **577**: 647-51.

12. Liang Z, Yao Y, Jiang B*, et al.* 3D printed graphene-based 3000 K probe. *Adv Funct Mater* 2021; **31**: 2102994.

13. Li T, Dong Q, Huang Z*, et al.* Interface engineering between multi-elemental alloy nanoparticles and a carbon support toward stable catalysts. *Adv Mater* 2022; **34**: 2106436.

14. Dong Q, Li T, Yao Y*, et al.* A general method for regenerating catalytic electrodes. *Joule* 2020; **4**: 2374-86.

15. Ping W, Wang R, Dong Q*, et al*. Printable, high-performance solid-state electrolyte films. *Sci Adv* 2020; **6**: eabc8641.

16. Mogera U, Kurra N, Radhakrishnan D*, et al.* Low cost, rapid synthesis of graphene on Ni: An efficient barrier for corrosion and thermal oxidation. *Carbon* 2014; **78**: 384-91.

17. Xu S, Zhong G, Chen C*, et al.* Uniform, scalable, high-temperature microwave shock for nanoparticle synthesis through defect engineering. *Matter* 2019; **1**: 759-69.

18. Zhong G, Xu S, Chen C*, et al.* Synthesis of Metal Oxide Nanoparticles by Rapid, High-Temperature 3D Microwave Heating. *Adv Funct Mater* 2019; **29**: 1904282.

19. Huang H, Zhou S, Yu C*, et al.* Rapid and energy-efficient microwave pyrolysis for high-yield production of highly-active bifunctional electrocatalysts for water splitting. *Energy Environ Sci* 2020; **13**: 545-53.

20. Zhong G, Xu S, Cui M*, et al.* Rapid, high-temperature, in situ microwave synthesis of bulk nanocatalysts. *Small* 2019; **15**: 1904881.

21. Fu Y, Rudnev AV, Wiberg GKH*, et al.* Single graphene layer on Pt(111) creates confined electrochemical environment via selective ion transport. *Angew Chem Int Ed* 2017; **56**: 12883-7.

22. Piner R, Li H, Kong X*, et al.* Graphene synthesis via magnetic inductive heating of copper substrates. *ACS Nano* 2013; **7**: 7495-9.

23. Seifert M, Drieschner S, Blaschke BM*, et al.* Induction heating-assisted repeated growth and electrochemical transfer of graphene on millimeter-thick metal substrates. *Diam Relat Mater* 2014; **47**: 46-52.

24. Wu Y, Wang K, Wei B*, et al.* Pyrolysis behavior of low-density polyethylene over HZSM-5 via rapid infrared heating. *Sci Total Environ* 2022; **806**: 151287.

25. Hu Q, Han Z, Wang X*, et al.* Facile synthesis of sub-nanometric copper clusters by double confinement enables selective reduction of carbon dioxide to methane. *Angew Chem Int Ed* 2020; **59**: 19054-9.

26. Liu S, Wang M, Ji H*, et al.* Altering the rate-determining step over cobalt single clusters leading to highly efficient ammonia synthesis. *Natl Sci Rev* 2020; **8**: nwaa136.

27. Ye W, Chen S, Lin Y*, et al.* Precisely tuning the number of Fe atoms in clusters on N-doped carbon toward acidic oxygen reduction reaction. *Chem* 2019; **5**: 2865-78.

28. Liu L, Zakharov DN, Arenal R*, et al.* Evolution and stabilization of subnanometric metal species in confined space by in situ TEM. *Nat Commun* 2018; **9**: 574.

29. Li L, Jiang Y-F, Zhang T*, et al.* Size sensitivity of supported Ru catalysts for ammonia synthesis: From nanoparticles to subnanometric clusters and atomic clusters. *Chem* 2021; **8**: 749-68.

30. Liu L, Lopez-Haro M, Lopes CW*, et al.* Regioselective generation and reactivity control of subnanometric platinum clusters in zeolites for high-temperature catalysis. *Nat Mater* 2019; **18**: 866-73.

31. Corma A, Concepción P, Boronat M*, et al.* Exceptional oxidation activity with size-controlled supported gold clusters of low atomicity. *Nat Chem* 2013; **5**: 775-81.

32. Li QY, Zhang L, Xu YX*, et al.* Smart Yolk/Shell ZIF-67@POM Hybrids as Efficient Electrocatalysts for the Oxygen Evolution Reaction. *ACS Sustainable Chem Eng* 2019; **7**: 5027-33.

33. Abdelkader-Fernández VK, Fernandes DM, Balula SS*, et al.* Advanced framework-modified POM@ZIF-67 nanocomposites as enhanced oxygen evolution reaction electrocatalysts. *J Mater Chem A* 2020; **8**: 13509-21.

34. Qin J-F, Xie J-Y, Wang N*, et al.* Surface construction of loose Co(OH)_2_ shell derived from ZIF-67 nanocube for efficient oxygen evolution. *J Colloid Interface Sci* 2020; **562**: 279-86.

35. Lin Y, Wang H, Peng C-K*, et al.* Co-Induced Electronic Optimization of Hierarchical NiFe LDH for Oxygen Evolution. *Small* 2020; **16**: 2002426.

36. Zhang Z, Tan Y, Zeng T*, et al.* Tuning the dual-active sites of ZIF-67 derived porous nanomaterials for boosting oxygen catalysis and rechargeable Zn-air batteries. *Nano Res* 2021; **14**: 2353-62.

37. Liu J, Wang C, Sun H*, et al.* CoOx/CoNy nanoparticles encapsulated carbon-nitride nanosheets as an efficiently trifunctional electrocatalyst for overall water splitting and Zn-air battery. *Appl Catal B Environ* 2020; **279**: 119407.

38. Li J-G, Sun H, Lv L*, et al.* Metal–Organic Framework-Derived Hierarchical (Co,Ni)Se_2_@NiFe LDH Hollow Nanocages for Enhanced Oxygen Evolution. *ACS Appl Mater Interfaces* 2019; **11**: 8106-14.

39. Ge H, Li G, Shen J*, et al.* Co4N nanoparticles encapsulated in N-doped carbon box as tri-functional catalyst for Zn-air battery and overall water splitting. *Appl Catal B Environ* 2020; **275**: 119104.

40. Liu S, Wang Z, Zhou S*, et al.* Metal–Organic-Framework-Derived Hybrid Carbon Nanocages as a Bifunctional Electrocatalyst for Oxygen Reduction and Evolution. *Adv Mater* 2017; **29**: 1700874.

41. Wang J, Gao D, Wang G*, et al.* Cobalt nanoparticles encapsulated in nitrogen-doped carbon as a bifunctional catalyst for water electrolysis. *J Mater Chem A* 2014; **2**: 20067-74.

42. Xie A, Zhang J, Tao X*, et al.* Nickel-based MOF derived Ni@NiO/N–C nanowires with core-shell structure for oxygen evolution reaction. *Electrochimi Acta* 2019; **324**: 134814.

43. Li Y, Jia B, Fan Y*, et al.* Bimetallic zeolitic imidazolite framework derived carbon nanotubes embedded with Co nanoparticles for efficient bifunctional oxygen electrocatalyst. *Adv Energy Mater* 2018; **8**: 1702048.

44. Yang Z, Zhao C, Qu Y*, et al.* Trifunctional self-supporting cobalt-embedded carbon nanotube films for ORR, OER, and HER triggered by solid diffusion from bulk metal. *Adv Mater* 2019; **31**: 1808043.

45. Liang Z, Zhang C, Yuan H*, et al.* PVP-assisted transformation of a metal–organic framework into Co-embedded N-enriched meso/microporous carbon materials as bifunctional electrocatalysts. *Chem Commun* 2018; **54**: 7519-22.

46. Han H, Paik JW, Ham M*, et al.* Atomic layer deposition-assisted fabrication of Co-nanoparticle/N-doped carbon nanotube hybrids as efficient electrocatalysts for the oxygen evolution reaction. *Small* 2020; **16**: 2002427.

47. Chen S, Duan J, Ran J*, et al.* N-doped graphene film-confined nickel nanoparticles as a highly efficient three-dimensional oxygen evolution electrocatalyst. *Energy Environ Sci* 2013; **6**: 3693-9.

48. Liang D, Zhang H, Ma X*, et al.* MOFs-derived core-shell Co_3_Fe_7_@Fe_2_N nanopaticles supported on rGO as high-performance bifunctional electrocatalyst for oxygen reduction and oxygen evolution reactions. *Mater Today Energy* 2020; **17**: 100433.

49. Ci S, Mao S, Hou Y*, et al.* Rational design of mesoporous NiFe-alloy-based hybrids for oxygen conversion electrocatalysis. *J Mater Chem A* 2015; **3**: 7986-93.

50. Feng X, Bo X, Guo L. CoM(M=Fe,Cu,Ni)-embedded nitrogen-enriched porous carbon framework for efficient oxygen and hydrogen evolution reactions. *J Power Sources* 2018; **389**: 249-59.

51. Tao Z, Wang T, Wang X*, et al.* MOF-derived noble metal free catalysts for electrochemical water splitting. *ACS Appl Mater Interfaces* 2016; **8**: 35390-7.

52. Feng Y, Yu X-Y, Paik U. N-doped graphene layers encapsulated NiFe alloy nanoparticles derived from MOFs with superior electrochemical performance for oxygen evolution reaction. *Sci Rep* 2016; **6**: 34004.

53. Tu Y, Ren P, Deng D*, et al.* Structural and electronic optimization of graphene encapsulating binary metal for highly efficient water oxidation. *Nano Energy* 2018; **52**: 494-500.

54. Yuan Q, Yu Y, Sherrell PC*, et al.* Fe/Co-based bimetallic MOF-derived Co_3_Fe_7_@NCNTFs bifunctional electrocatalyst for high-efficiency overall water splitting. *Chem Asian J* 2020; **15**: 1728-35.

55. Khani H, Grundish NS, Wipf DO*, et al.* Graphitic-shell encapsulation of metal electrocatalysts for oxygen evolution, oxygen reduction, and hydrogen evolution in alkaline solution. *Adv Energy Mater* 2020; **10**: 1903215.

56. Wang G, Chang J, Koul S*, et al.* CO_2_ bubble-assisted Pt exposure in PtFeNi porous film for high-performance zinc-air battery. *J Am Chem Soc* 2021; **143**: 11595-601.

57. Le Formal F, Yerly L, Potapova Mensi E*, et al.* Influence of composition on performance in metallic iron–nickel–cobalt ternary anodes for alkaline water electrolysis. *ACS Catal* 2020; **10**: 12139-47.

58. Wang X, Xiao H, Li A*, et al.* Constructing NiCo/Fe_3_O_4_ Heteroparticles within MOF-74 for Efficient Oxygen Evolution Reactions. *J Am Chem Soc* 2018; **140**: 15336-41.

59. Gupta S, Qiao L, Zhao S*, et al.* Highly active and stable graphene tubes decorated with FeCoNi alloy nanoparticles via a template-free graphitization for bifunctional oxygen reduction and evolution. *Adv Energy Mater* 2016; **6**: 1601198.

60. Gupta S, Zhao S, Wang XX*, et al.* Quaternary FeCoNiMn-based nanocarbon electrocatalysts for bifunctional oxygen reduction and evolution: promotional role of Mn doping in stabilizing carbon. *ACS Catal* 2017; **7**: 8386-93.

61. Lu Y, Huang K, Cao X*, et al.* Atomically dispersed intrinsic hollow sites of *M-M_1_-M* (*M_1_* = Pt, Ir; M = Fe, Co, Ni, Cu, Pt, Ir) on FeCoNiCuPtIr nanocrystals enabling rapid water redox. *Adv Funct Mater* 2022; **32**: 2110645.

62. Ding Z, Bian J, Shuang S*, et al.* High entropy intermetallic–oxide core–shell nanostructure as superb oxygen evolution reaction catalyst. *Adv Sustainable Syst* 2020; **4**: 1900105.

63. Liu L-H, Li N, Han M*, et al.* Scalable synthesis of nanoporous high entropy alloys for electrocatalytic oxygen evolution. *Rare Met* 2022; **41**: 125-31.

64. Jin Z, Lyu J, Zhao Y-L*, et al.* Rugged high-entropy alloy nanowires with in situ formed surface spinel oxide as highly stable electrocatalyst in Zn–air batteries. *ACS Mater Lett* 2020; **2**: 1698-706.

65. Fang G, Gao J, Lv J*, et al.* Multi-component nanoporous alloy/(oxy)hydroxide for bifunctional oxygen electrocatalysis and rechargeable Zn-air batteries. *Appl Catal B- Environ* 2020; **268**: 118431.

66. Tang J, Xu JL, Ye ZG*, et al.* Microwave sintered porous CoCrFeNiMo high entropy alloy as an efficient electrocatalyst for alkaline oxygen evolution reaction. *J Mater Sci Technol* 2021; **79**: 171-7.

67. Wang B, Wang C, Yu X*, et al.* General synthesis of high-entropy alloy and ceramic nanoparticles in nanoseconds. *Nat Synth* 2022; **1**: 138-46.
